# Preeclampsia is Associated With Reduced *ISG15* Levels Impairing Extravillous Trophoblast Invasion

**DOI:** 10.3389/fcell.2022.898088

**Published:** 2022-06-28

**Authors:** Asli Ozmen, Ozlem Guzeloglu-Kayisli, Selcuk Tabak, Xiaofang Guo, Nihan Semerci, Chinedu Nwabuobi, Kellie Larsen, Ali Wells, Asli Uyar, Sefa Arlier, Ishani Wickramage, Hasan Alhasan, Hana Totary-Jain, Frederick Schatz, Anthony O. Odibo, Charles J. Lockwood, Umit A. Kayisli

**Affiliations:** ^1^ Department of Obstetrics and Gynecology, Morsani College of Medicine, University of South Florida, Tampa, FL, United States; ^2^ Department of Obstetrics, Gynecology and Reproductive Sciences, Yale University School of Medicine, New Haven, CT, United States; ^3^ Department of Molecular Pharmacology and Physiology, Morsani College of Medicine, University of South Florida, Tampa, FL, United States; ^4^ Divisions of Maternal-Fetal Medicine and Ultrasound, Department of Obstetrics and Gynecology, Washington University School of Medicine, St. Louis, MO, United States

**Keywords:** *ISG15*, *IL6*, IL11, preeclampsia, extravillous trophoblast, differentiation, invasion, inflammation

## Abstract

Among several interleukin (IL)-6 family members, only IL-6 and IL-11 require a gp130 protein homodimer for intracellular signaling due to lack of intracellular signaling domain in the IL-6 receptor (IL-6R) and IL-11R. We previously reported enhanced decidual IL-6 and IL-11 levels at the maternal-fetal interface with significantly higher peri-membranous IL-6 immunostaining in adjacent interstitial trophoblasts in preeclampsia (PE) vs. gestational age (GA)-matched controls. This led us to hypothesize that competitive binding of these cytokines to the gp130 impairs extravillous trophoblast (EVT) differentiation, proliferation and/or invasion. Using global microarray analysis, the current study identified inhibition of interferon-stimulated gene 15 (*ISG15*) as the only gene affected by both IL-6 plus IL-11 vs. control or IL-6 or IL-11 treatment of primary human cytotrophoblast cultures. *ISG15* immunostaining was specific to EVTs among other trophoblast types in the first and third trimester placental specimens, and significantly lower *ISG15* levels were observed in EVT from PE vs. GA-matched control placentae (*p* = 0.006). Induction of primary trophoblastic stem cell cultures toward EVT linage increased *ISG15* mRNA levels by 7.8-fold (*p* = 0.004). *ISG15* silencing in HTR8/SVneo cultures, a first trimester EVT cell line, inhibited invasion, proliferation, expression of *ITGB1* (a cell migration receptor) and filamentous actin while increasing expression of *ITGB4* (a receptor for hemi-desmosomal adhesion). Moreover, *ISG15* silencing further enhanced levels of IL-1β-induced pro-inflammatory cytokines (*CXCL8*, *IL-6* and *CCL2*) in HTR8/SVneo cells. Collectively, these results indicate that *ISG15* acts as a critical regulator of EVT morphology and function and that diminished *ISG15* expression is associated with PE, potentially mediating reduced interstitial trophoblast invasion and enhancing local inflammation at the maternal-fetal interface. Thus, agents inducing *ISG15* expression may provide a novel therapeutic approach in PE.

## Introduction

Preeclampsia (PE), a major cause of excess maternal and perinatal morbidity and mortality, has a worldwide prevalence of 2%–8% (Steegers et al., 2010; Huppertz, 2018). Paracrine interactions among trophoblasts, decidual cells, and maternal immune cells in the decidua generate the moderate inflammatory milieu required for sufficiency of trophoblast invasion during normal implantation and placentation (Redman et al., 1999; Schatz et al., 2016). However, excess inflammation with immune maladaptation in the maternal decidua causes shallow trophoblast invasion, resulting in incomplete spiral artery transformation characteristic of PE (Aneman et al., 2020; Garrido-Gomez et al., 2020).

Decreased anti-inflammatory cytokine expression e.g., interleukin-4 (IL-4), IL-10 (Harmon et al., 2016) and elevated pro-inflammatory cytokine levels e.g., IL-1β, IL-8 have been reported in maternal serum, decidua, umbilical cord and chorionic villi in PE (Tosun et al., 2010; Bellos et al., 2018; Aggarwal et al., 2019). Treatment with IL-11 induces *in vitro* human decidualization (Dimitriadis et al., 2002) while IL-11Rα deficient mice experience implantation failure (Robb et al., 1998), confirming the physiologic role of IL-11 in the establishment of normal pregnancy. In contrast, IL-6 deficient mice have normal fertilization and implantation indicating no critical role of IL-6 during early pregnancy (Prins et al., 2012; Sakurai et al., 2012). Enhanced maternal serum levels of IL-6 (Xiao et al., 2012; Zhang et al., 2013) and IL-11 (Winship et al., 2015) were reported in PE. Moreover, we have previously observed that in PE vs. gestational age (GA)-matched controls, decidual IL-6 and IL-11 levels are significantly elevated and interstitial extravillous trophoblasts (EVTs) adjacent to the decidual cells display peri-membranous immunoreactivity for both IL-11 and IL-6 (Lockwood et al., 2008; Basar et al., 2010). We also found that peri-membranous IL-6, but not IL-11 immunoreactivity is significantly higher in EVTs from PE vs. control placentas (Lockwood et al., 2008; Basar et al., 2010). These results suggest that a paracrine co-regulation of EVT function(s) by decidual cell derived IL-6 and IL-11 since both cytokines bind to a gp130 homodimer as the common receptor to initiate intracellular signaling among several IL-6 family members (Dahmen et al., 1998). IL-6 and lL-11 have different affinities for their own receptors and for the common gp130 receptor. The affinity (Kd) of IL-6 for its IL-6R is ∼1 nM range, whereas the affinity of the *IL6*-*IL6*R complex for gp130 is 100 times higher (Rose-John, 2012) which means a Kd of ∼10 pM. The Kd of IL-11 for its IL-11R is ∼10 nM, whereas the Kd of the IL11-IL11R complex for gp130 is between 300 and 800 pM (Reactome, 2022). Thus, the IL-6-*IL6*R GP130 complex has the highest affinity. Thus, we hypothesize that competitive binding of IL-6 vs. IL-11 to gp130 homodimer in trophoblasts can trigger PE by impairing interstitial trophoblast differentiation, invasion, and/or inflammatory responses.

The current study focused on the impact of separate vs. combined IL-6 and IL-11 treatments in primary trophoblast cultures using whole genome microarray analysis. This initial microarray analysis identified down-regulation of interferon-stimulated gene 15 (*ISG15*) as the only common gene in IL-6 plus IL-11 co-treatment vs. separate treatment with either IL-6 or IL-11. *In situ* analysis of placental specimens revealed predominant *ISG15* expression by EVTs compared to other trophoblast types, and significantly lower *ISG15* levels in EVTs from PE vs. gestational age (GA)-matched controls. Moreover, *in vitro* analyses identified *ISG15* as a critical regulator of trophoblast differentiation, invasion, and pro-inflammatory cytokine expression.

## Materals and Methods

### Placental Specimens

Previously banked placental paraffin specimens were used after approval by the University of South Florida Institutional Review Board (#19472). Written informed consent was received from patients prior to inclusion in the study. Placental specimens containing decidua basalis were obtained from PE and GA-matched idiopathic preterm birth or uncomplicated pregnancies with scheduled repeated cesarean deliveries. As we described previously (Canfield et al., 2019), criteria for PE diagnosis included a blood pressure >140 mmHg systolic or >90 mmHg diastolic accompanied by proteinuria after 20 weeks of gestation. To determine cell types expressing *ISG15* at the maternal fetal interface during early pregnancy, paraffin sections were also obtained from previously banked first trimester placental specimens after voluntary termination of uncomplicated pregnancy.

### Primary Trophoblast Cell Cultures

Isolation of cytotrophoblasts (CTBs) from term placental specimens obtained from cesarean delivery without labor was performed as described (Tang et al., 2011; Guzeloglu-Kayisli et al., 2020). Briefly, after removal of decidual tissues, fragments of chorionic villi were subjected to enzymatic digestion in a Hanks’ Balanced Salt Solution (Invitrogen, Carlsbad, CA) solution containing 0.25% trypsin (Invitrogen), 0.2% DNase I (Roche, Indianapolis, IN), 25 mM HEPES, 2 mM CaCl_2_ and 0.8 mM MgSO_4_ in at 37°C for 90 min. Cell suspension were then separated using a discontinuous (30%, 35%, 45%, 50%) Percoll gradient (GE Healthcare, Piscataway, NJ). Cells in 35%–45% Percoll interface were collected and immune-purified using mouse anti-human CD9 (R&D Inc., Minneapolis, MN) and mouse anti-CD45 (R&D) antibodies for negative selection by removing fibroblasts and immune cells. Then, the unbound cells were collected, washed, and cultured in 6-well plates in DMEM/F12 (Gibco, Life Technologies Corporation, Grand Island, NY) containing 10% FBS (Gemini Bio Products, West Sacramento, CA). Following 24 h incubation in serum-free media, cells were treated with vehicle or recombinant human IL-11 (10 ng/ml, R&D) or IL-6 (10 ng/ml, R&D) or IL-11 + IL-6 for 6 h for microarray analysis (Monzon-Bordonaba et al., 2002; Monhasery et al., 2016; Strickland et al., 2017)

### HTR8/SVneo and JEG3 Cell Cultures

HTR8/SVneo (a first trimester EVT cell line) and JEG3 (a choriocarcinoma cell line) cultures (ATCC, Manassas, VA) were grown in DMEM/F12 (1:1) (Gibco) supplemented 5% FBS (Gemini Bio) and 1% antibiotic and antimycotics (Gibco). Confluent cells were trypsinized and seeded in 6-well plates (10^6^ cells/well). Following 24 h incubation in serum-free media, cells were treated with recombinant human interferon (IFN)γ (R&D) at 10 and 100 ng/ml concentration for 6 h then washed with ice-cold PBS and stored at -80°C for RNA isolation.

### Microarray Analysis

Total RNA from CTBs cultures was isolated using RNeasy Mini isolation kit and cleaned up with RNeasy MinElute Cleanup Kit according to the manufacturer’s instructions (Qiagen, Valencia, CA). The quality of RNA was confirmed by a Bio-Rad bioanalyzer. Samples were submitted to W. M. Keck Foundation Biotechnology Resource Laboratory at Yale University for whole genome microarray analysis using Illumina Human HT-12 v4 Expression Bead Chip Kit (Illumina Inc., San Diego, CA) per manufacturer’s instructions. GeneSpring GX12.5 software (Agilent Technologies, Redwood City, CA) were used to analyze raw data without normalization. Normalization was performed as described (Guzeloglu-Kayisli et al., 2014). Genes with a fold change of > 1.5 increased or decreased expression levels and a *p*-value of < 0.05 were considered differentially expressed.

### Immunohistochemistry

Deparaffinization of placental sections was followed by heat-induced antigen retrieval using 10 mM citrate buffer (pH 6.0) and endogenous peroxidase quenching using 3% hydrogen peroxide. After several washes with Tris-buffered saline (TBS, pH 7.2), slides were blocked with 5% normal horse serum (Vector Labs, Burlingame, CA) for 30 min at room temperature, then incubated overnight at 4°C with mouse anti-*ISG15* antibody (1:1250, Santa Cruz Biotechnology, Inc., Dallas, TX). After washes in TBS, the slides were incubated with biotinylated horse anti-mouse secondary antibody (1:400, Vector Labs) for 30 min, and signals were enhanced by using streptavidin-conjugated peroxidase complex kit (Vector Labs.) for 30 min. Chromogen DAB (3,3′-diaminobenzidine) and Mayer’s hematoxylin (Vector Labs.) were used to develop immunoreactivity and nuclear counterstaining, respectively. A second set of serial sections were double immunostained sequentially for mouse anti-cytokeratin-7 (1:600, DAKO, Carpinteria, CA, United States) and chicken anti-vimentin (1:400, Abcam, Cambridge, MA, United States) antibodies to identify trophoblasts and decidual cells, respectively, as described (Sinkey et al., 2020). DAB (brown) for cytokeratin and Vector RED for vimentin (Vector Labs.) were used as chromogens. For negative controls, appropriate normal IgG isotypes were used at the same concentration of each corresponding primary antibody. Immunoreactivity for *ISG15* was assessed by histological scoring (HSCORE), a semi-quantitative method that evaluates the intensity and the number of immunostained cells by two blinded investigators as described (Lockwood et al., 2012) using Axio Imager-A2 light microscope (Zeiss; Oberkochen, Germany) and ZEN imaging system (Zeiss).

### Culture of Trophoblast Stem Cells and Induction of EVT Differentiation

Human trophoblast stem cells (TS) derived from first trimester placentas were generated by Drs. Okae and Arima (Okae et al., 2018) and were provided by RIKEN BRC through the National Bio-resource Project of the Mext/AMED, Japan. TS cells are cultured and induced to EVT (TS-EVT) differentiation as described (Okae et al., 2018).

### Small Interfering RNA (siRNA) Transfection

HTR8/SVneo cells were plated in 6-well or 96-well culture plates or 8-well chamber slides for silencing of *ISG15* expression. The following day, cells were transiently transfected with either *ISG15*-specific or non-specific scrambled control siRNA (Santa Cruz) in Opti-MEM media (Invitrogen) using Lipofectamine RNAiMAX Reagent (Invitrogen) according to the manufacturer’s instructions. After 44 h, cells in 6-well plate were treated with vehicle or recombinant human IL-1β (10 ng/ml, R&D) for 6 h, then washed with ice-cold PBS and stored at −80°C for RNA isolation. Transfection efficiency was controlled by qPCR and Western blotting.

### Western Blot Analysis

Tissue lysates obtained from PE and GA-matched control placental samples containing decidua basalis (*n* = 4/each group) as well as cell lysates obtained from control- and *ISG15*-siRNA transfected HTR8/SVneo cells subjected to SDS-PAGE (Bio-Rad, Hercules, CA, United States), and transferred onto a nitrocellulose membrane. Membranes were first blocked with 5% non-fat dry milk in TBS with 0.1% Tween 20 (TBS-T) for 1 h at room temperature, then incubated with mouse-anti-*ISG15* (1:500; Santa Cruz) overnight at 4°C. Several washing steps of membranes followed incubation with peroxidase-conjugated anti-mouse secondary antibody (1:5000; Vector Labs) for 1 h at room temperature. An enhanced chemiluminescence kit (Thermo Fisher Scientific) was used to detect immunoblot bands. After stripping, the membrane was re-probed with HLA-G (1:1000: Cell signaling) overnight at 4°C followed by 1 h room temperature peroxidase-conjugated anti-rabbit secondary antibody (1:5000; Vector Labs) incubation. For endogenous loading control, membranes were then re-probed with HRP-conjugated rabbit-anti-β-actin (1:1000; Cell Signaling) antibody for 30 min at room temperature. Densitometric quantification was performed *via* Image J software (ImageJ 1.52a. National Institutes of Health, Bethesda, MD).

### Quantitative Real Time-PCR (qPCR)

Total RNA from cultured TS, TS-EVTs, JEG3 and HTR8/SVneo cells were isolated by using RNeasy Mini isolation kit (Qiagen) according to the manufacturer’s instructions. 500 ng total RNA was reverse transcribed by using the Retroscript RT (Invitrogen). qPCR was performed with Taqman gene expression assays (Applied Biosystems, Foster City, CA,United States) for human *ISG15* (Hs00192713_m1), *IL1B* (Hs01555410_m1), C-X-C Motif Chemokine Ligand 8 (*CXCL8* (=IL8); Hs00174103_m1), *IL6* (Hs00985639_m1), C-C Motif Chemokine Ligand 2 (*CCL2*; Hs00234140_m1), integrin-β4 (*ITGB4*; Hs00236216_m1), integrin-β1 (*ITGB1*; Hs01127536_m1), *HLA-G* (Hs00365950_g1), *TP63* (Hs00978340_m1) and the reference genes β-actin (*ACTB*; Hs99999903_m1) and *GAPDH* (Hs02758991_g1). Samples were run in duplicate and average mean C_T_ value used for each sample. Relative quantification was calculated using 2^−ΔΔCT^ method and reported as fold change.

### Fluorescence Labelling of Filamentous Actin (F-Actin)

Control- and *ISG15*-siRNA transfected HTR8/SVneo cells seeded onto 8-well culture slides (BD biosciences, Bedford, MA, United States) and were fixed with 4% paraformaldehyde for 20 min at 4^°^C. After washing and permeabilization steps, the slides were incubated with TRITC-conjugated phalloidin (Millipore, San Diego, CA, United States), which preferentially binds to actin filaments, for 30 min at room temperature. After washing, the slides were treated with 6-diamino-2-phenylindole (DAPI) for 10 min and mounted with antifade mounting medium (Vector Lab). Slides were examined under Axio Observer-Z1 immunofluorescence/phase-contrast microscope (Zeiss) with ZEN Imaging system (Zeiss).

### Wound Healing Assay

Either control or *ISG15* siRNA forward transfected HTR8/SVneo cells (2 × 10^4^ cells per insert) were seeded into 35 mm culture dishes via 2-well silicone inserts with 500 µm gap (Ibidi United States, Inc., Fitchburg, Wisconsin) according to the manufacturer’s protocol. Twenty-4 h later, insert was removed, and cells were monitored at 0, 4, 18, 24, 44th h under Axio Observer-Z1 immunofluorescence/phase contrast microscope (Zeiss).

### Migration and Invasion Assays

Transwell cell migration and invasion assays were performed using a CytoSelectTM 24-well non-coated and Matrigel-coated transwell with 8-µm pore size polycarbonate filters, respectively (Cell Biolabs Inc., San Diego, CA, United States). In both assays, control- or *ISG15*-siRNA transfected HTR8/SVneo cells (5 × 10^5^ cells per well) were suspended in DMEM/F12 media with 1% FBS and seeded in the upper chamber of a transwell insert that was placed in the lower chamber containing DMEM/F12 media with 10% FBS. Forty-eight or 60 h later, non-migrated or invaded cells, respectively were removed from the upper chamber. The migrated or invaded cells were colorimetrical quantified by spectrophotometric readings at 560 nm according to the manufacturer’s protocol (Cell Biolabs).

### Cell Proliferation and Apoptosis Assay

HTR8/SVneo cells plated into 96-wells (1 × 10^4^ cells) were transiently transfected with either control or *ISG15* siRNA. Proliferation was measured using Bromodeoxyuridine (BrdU) cell proliferation kit (Cell Signaling) as described (Kayisli et al., 2015). Absorbance was measured at 450 nm using Spectra Max190 microplate reader (Molecular Devices LLC, San Jose, CA, United States) to detect BrdU incorporation in each well. Apoptosis index was determined in a second set of experiments using an ELISA-based cell death detection kit (Roche Diagnostics, Indianapolis, IN) as described in (Kayisli et al., 2015) and colorimetric reaction measured at 405 nm.

### Enzyme-Linked Immunosorbent Assay (ELISA)

*ISG15* ELISA is performed according to the manufacturer’s instructions (Elabscience, Houston, TX) in 1^st^, second and 3^rd^ trimester maternal serum samples, collected from women who developed PE (*n* = 7) in later stages of pregnancy or from gestational age matched women without pregnancy complications (control, *n* = 7). IL-6 and MCP-1 protein secretion levels were measured using ELISA assay in culture media collected from control vs. *ISG15*-siRNA transfected HTR8/SVneo cells treated with 10 ng/ml IL-1β for 6 h according to manufacturer’s instructions (R&D Systems).

### Statistical Analysis

Results with normal distribution were analyzed by Student’s *t-test* for two group comparison or One-Way Analysis of Variance followed by Holm-Sidak method for multiple comparisons. Data not normally distributed were analyzed by Kruskal-Wallis One Way Analysis of Variance on Ranks followed by Student-Newman-Keuls method. Statistical analysis was performed using Sigma Plot version 11.0 (Systat Software Inc.); *p < 0.05* was considered statistically significant.

## Results

### Impacts of IL-11 and IL-6 Co-Treatment on Global Transcription Repertoire of CTB Cultures

**FIGURE 1:**
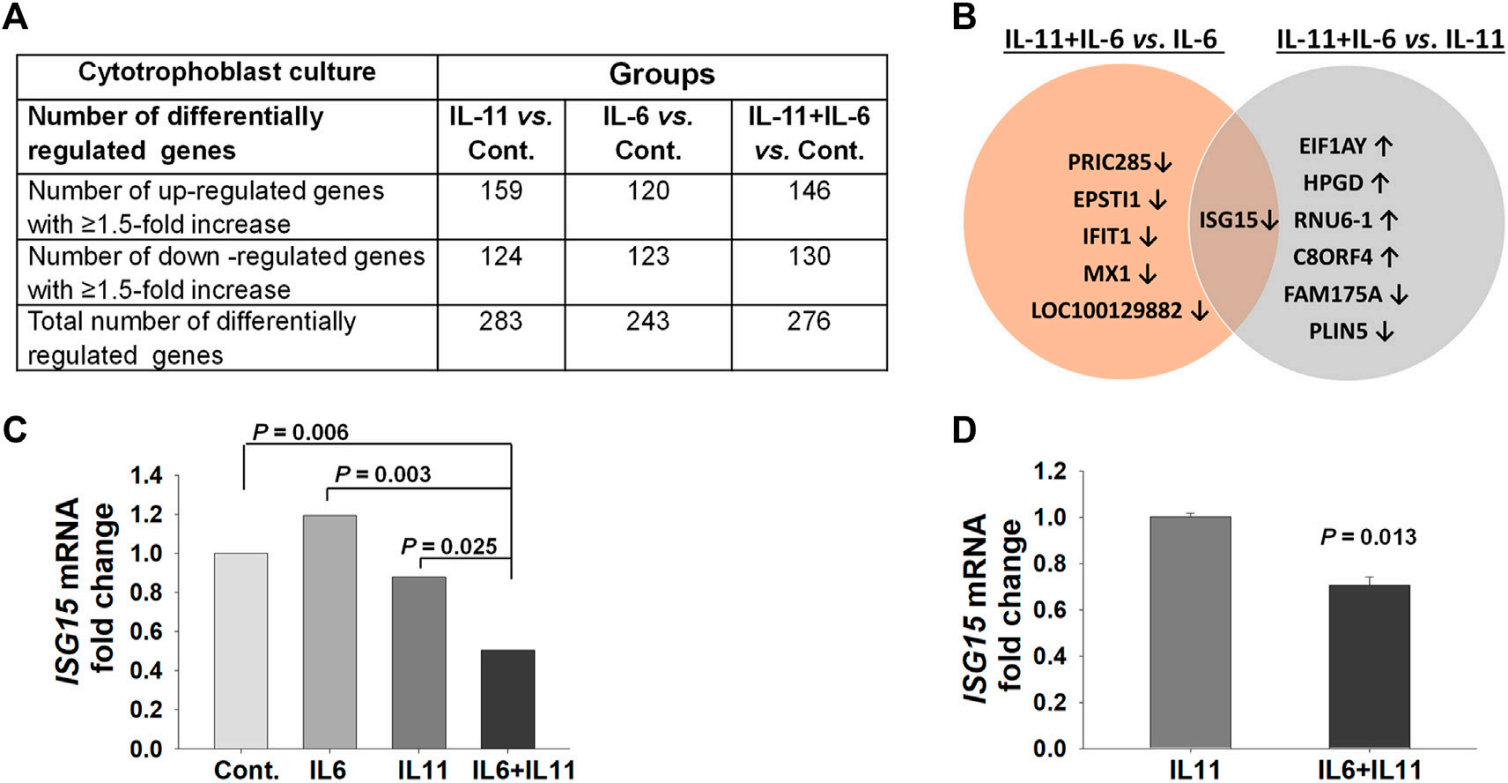
Microarray analysis identifies the *ISG15* transcript as a common, differentially regulated, gene in primary cytotrophoblast cultures co-treated with IL-11 and IL-6. **(A)** Number of genes differentially regulated by treatment with either IL-11 (10 ng/ml) or IL-6 (10 ng/ml) or IL-11 + IL-6 vs. control (Cont.) in cytotrophoblast cell cultures for 6 h. **(B)** Genes differentially regulated by IL-11 + IL-6 co-treatment versus treatment with IL-6 or IL-11 separately. **(C)** Graph represents fold changes in pair-comparison of microarray data for *ISG15* mRNA in control, IL-6, and IL-11 vs. IL-11 + IL-6 treatment groups (*n* = 3). **(D)** *ISG15* mRNA expression levels by RT-qPCR analysis; bars represent Mean ± SEM and compared by using *t*-test, *n* = 3 with triplicates for each experiment.

To mimic the separate and combined paracrine effects of IL-11 and IL-6 on trophoblasts in normal versus PE pregnancies, primary CTB cultures treated with IL-11 or IL-6 or IL-11 + IL-6 were analyzed by whole genome microarray. These microarray analyses revealed that IL-11 treated CTBs displayed a total of 283 differentially expressed genes (159 of which are upregulated and 124 are downregulated), whereas IL-6 treated CTBs displayed a total of 243 differentially expressed genes (120 are upregulated and 123 are downregulated) compared control group (Figure 1A and Supplementary Table S1). However, IL-11 + IL-6 co-treated CTBs exhibited 276 differentially expressed genes (146 of which are upregulated and 130 are downregulated) compared to control group (Figure 1A, Supplementary Table S1 *)*.

Since IL-11 and IL-6 can compete to activate target cells through binding their shared receptor, the global transcription repertoire of CTB cultures treated with IL-11 + IL-6 was compared to those treated with either IL-11 or IL-6. A total of seven genes are differentially regulated by IL-11 + IL-6 co-treatment vs. treatment with IL-11 alone (four of which are upregulated and three downregulated), whereas compared to IL-6 alone, a total of six genes are differentially regulated by IL-6 + IL-11 (all of which are downregulated, Figure 1B). Among all the comparisons, *ISG15* was the only gene expression common to IL-11 + IL-6 treatment and was downregulated (Figures 1B,C **)**. Analysis using q-PCR confirmed that *ISG15* mRNA expression is significantly downregulated by IL-11 + IL-6 versus treatment of IL-11 alone in primary CTB (Figure 1D).

### *ISG15* Expression is Specific to EVTs and Lower in Preeclamptic Placentas

**FIGURE 2:**
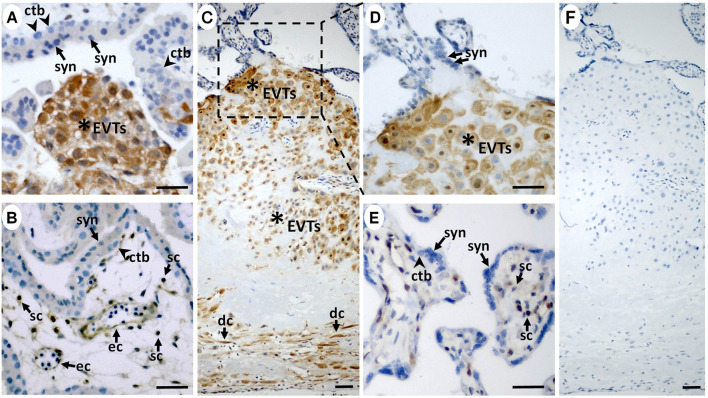
*ISG15* protein is preferentially expressed by extravillous trophoblasts during ***in vivo*** differentiation. In first (**A,B**; *n* = 2) and third trimester (**C–E**; *n* = 11) placentas, immunoreactive *ISG15* expression was predominantly limited to EVTs (stars) with a moderate expression in some villous stromal cells (sc) and endothelial cells (ec) in first **(B)** and third trimester **(E)** placental samples. **(C)** Decidual cells (dc) were also positive for *ISG15* immunoreactivity. **(F)** Negative control staining displayed no immunoreactivity. Scale bars = 30 µm. (syn = syncytiotrophoblast, ctb = cytotrophoblast).

**FIGURE 3:**
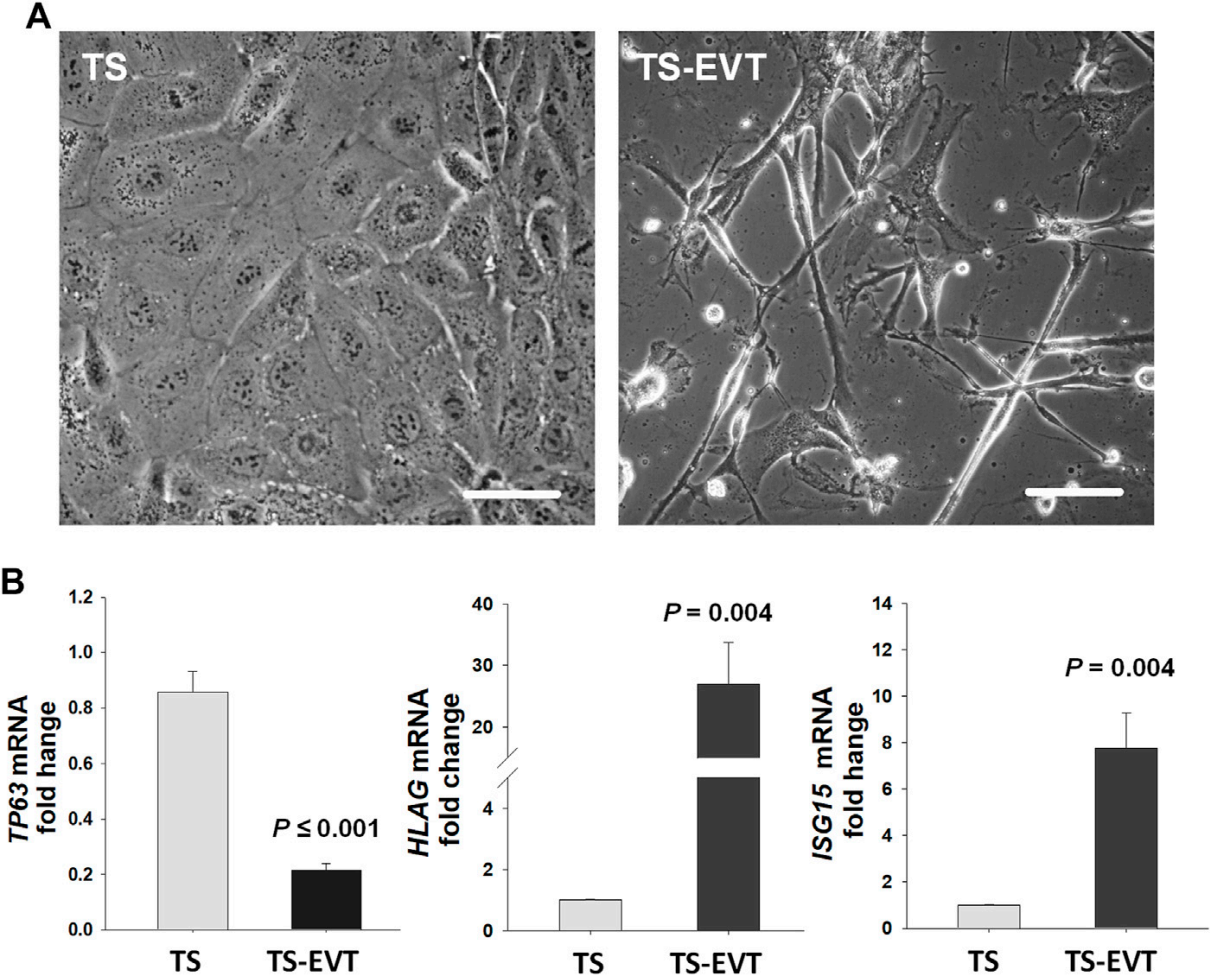
***In vitro*** differentiation of trophoblastic stem cells into extravillous trophoblasts significantly enhance *ISG15* mRNA levels. **(A)** Cultured trophoblastic stem (TS) cells display polygonal shapes with tight cell-cell attachments whereas TS induced extravillous trophoblastic (TS-EVT) cells display elongated cell morphology with multiple protrusion and less cell-cell contacts. **(B)** *TP63*, *HLAG*, and *ISG15* mRNA levels in TS vs. TS-EVT cells. Bars represent Mean ± SEM and compared by using *t*-test. TS cells (*n* = 6), TS-EVT cells (*n* = 5). Scale bars = 30 µm.

We then examined *ISG15* immunostaining in first and third trimester placental sections to determine cell type(s) expressing *ISG15* as well as its gestational age-related expression pattern. *ISG15* immunoreactivity was specifically observed in EVTs in both the first (Figures 2A,B) and third trimesters (Figures 2C,D), with no expression in villous CTBs and syncytiotrophoblast. Some stromal and endothelial cells in villi (Figures 2B,E), and decidual cells at the maternal-fetal interface also displayed *ISG15* immunoreactivity (Figure 2C). In order to confirm *ISG15* expression is associated with EVT differentiation, we used a previously established cultures of TS cells, isolated from first trimester CTBs that could be differentiated into EVT like cells (Okae et al., 2018). In comparison with TS cell cultures, induction of TS toward EVT differentiation (TS-EVT) is confirmed with their altered cellular morphology (Figure 3A **)** and decreased *TP63* mRNA and increased *HLA-G* mRNA expression and resulted in ∼8-fold increase in *ISG15* expression in TS- EVTs (Figure 3B).

**FIGURE 4:**
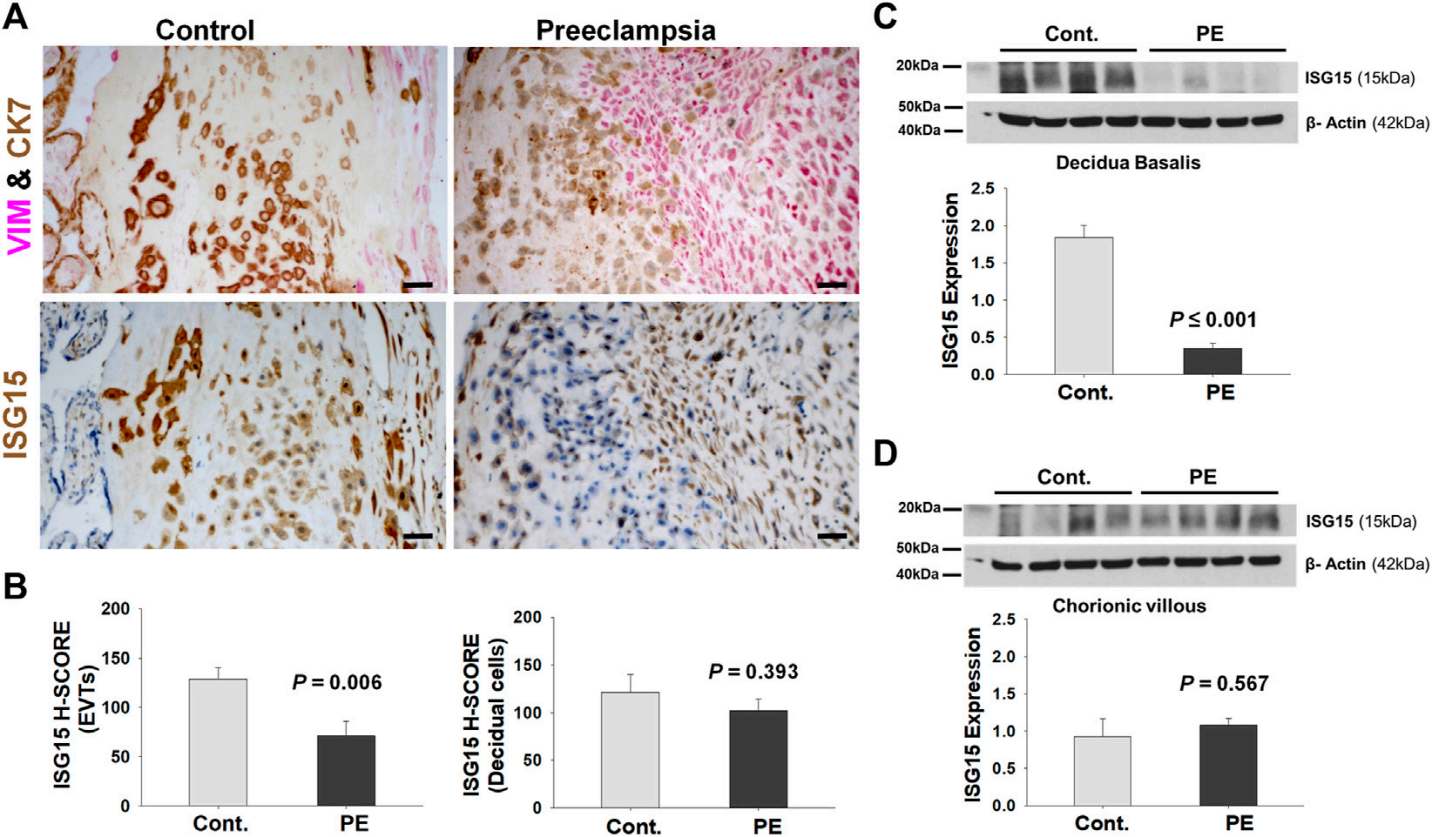
EVTs display diminished *ISG15* expression in preeclamptic placenta. **(A)** *ISG15* immunoreactivity in EVTs and decidual cells in vimentin (VIM, red, a decidual cell marker) - cytokeratin7 (CK7, brown, a trophoblast marker) double immuno-stained serial sections of control (Cont, *n* = 11) vs. preeclamptic (PE, *n* = 11) placentas. **(B)** H-SCORE of *ISG15* immunoreactivity in EVTs and decidual cells. **(C,D)** Western blot analysis of decidua basalis (**C;** *n* = 4/group) and chorionic villi samples (**D**; *n* = 4/group) showing *ISG15* expression in PE vs. control specimens. Bars represent Mean ± SEM and compared by using *t*-test; Scale bars = 30 µm.

Next, we compared *ISG15* expression levels in PE versus GA-matched controls. EVTs and decidual cells at the maternal-fetal interface were identified by double-immunostaining of cytokeratin 7 (trophoblast marker) and vimentin (decidual cells marker; Figure 4A). Evaluation of the maternal-fetal interface by *ISG15* H-SCORE analysis revealed that compared to controls, EVTs in PE placentas had significantly lower *ISG15* immunoreactivity (128.8 ± 11.5 vs. 71.2 ± 14.7, *p =* 0.006, Figure 4B). In contrast, decidual cells displayed similar *ISG15* expression between groups (Cont. vs. PE; 121.5 ± 18.4 vs. 102.1 ± 12.4, *p =* 0.393, Figure 4B). Western blot analysis was also performed in placental samples which were separated into two parts: chorionic tissues, which contain placental villi, and the decidual tissues, which contain decidua basalis with possible minor contamination of placental villi. Western blot analysis of these tissues revealed that *ISG15* expression was significantly decreased in decidua basalis samples from PE placentas compared to controls (0.35 ± 0.07 vs 1.84 ± 0.16, *p ≤* 0.001; Figure 4C), whereas no difference in *ISG15* expression was found in chorionic villi between groups (Figure 4D). Besides villous cytotrophoblasts, the chorionic villi include extravillous trophoblasts which reside in the chorionic plate, trophoblastic cell columns/islands and placental fibrinoid deposits (Baergen, 2011). Therefore, *ISG15* expression detected in microarray analysis of CTBs and in immunoblots of chorionic villous lysates predominantly represents *ISG15* expression in EVT cell population.

To confirm that reduced *ISG15* level is not due to reduced EVT cell number in decidua basalis, we assessed HLA-G levels in the same samples and observed similar HLA-G protein levels between groups (Supplementary Figure S1). Given *ISG15* is also a secretory protein that can be detected in serum (Alcala et al., 2020), we then performed ELISA analysis to evaluate whether there is a reduction in the serum *ISG15* levels reflective of what we observed in preeclamptic EVTs at the maternal-fetal interface. We analyzed *ISG15* levels in 1st, second and 3rd trimester maternal blood serum samples from women who developed later onset PE and age matched controls without identification of a significant difference between groups at any gestational age (Supplementary Figure S2).

### *ISG15* siRNA Treatment Affects HTR8/SVneo Cell Morphology

To uncover specific role(s) of *ISG15* in CTB functions, we first examine two trophoblastic cell lines JEG3 and HTR8/SVneo cells, to determine most appropriate one for further experiments. In comparison with vehicle (control) treatment, 10 and 100 ng/ml of IFNγ treatments significantly increased *ISG15* mRNA expression both in JEG3 cells (Cont. vs. 10 ng/ml IFNγ or 100 ng/ml IFNγ: 1.03 ± 0.05 vs. 4.00 ± 0.06, or 6.05 ± 0.08, *p ≤* 0.001, Supplementary Figure S3) and HTR8/SVneo cells (Cont. vs. 10 ng/ml IFNγ or 100 ng/ml IFNγ: 1.07 ± 0.07 vs. 15.84 ± 0.04, or 21.64 ± 0.13, *p ≤* 0.001, Supplementary Figure S3). HTR8/SVneo cell line was chosen for further experiments because it displayed a stronger IFNγ mediated *ISG15* response compared to JEG3 cell line, and it has a first trimester origin with invasive EVT phenotype (Jeyarajah et al., 2019).

**FIGURE 5:**
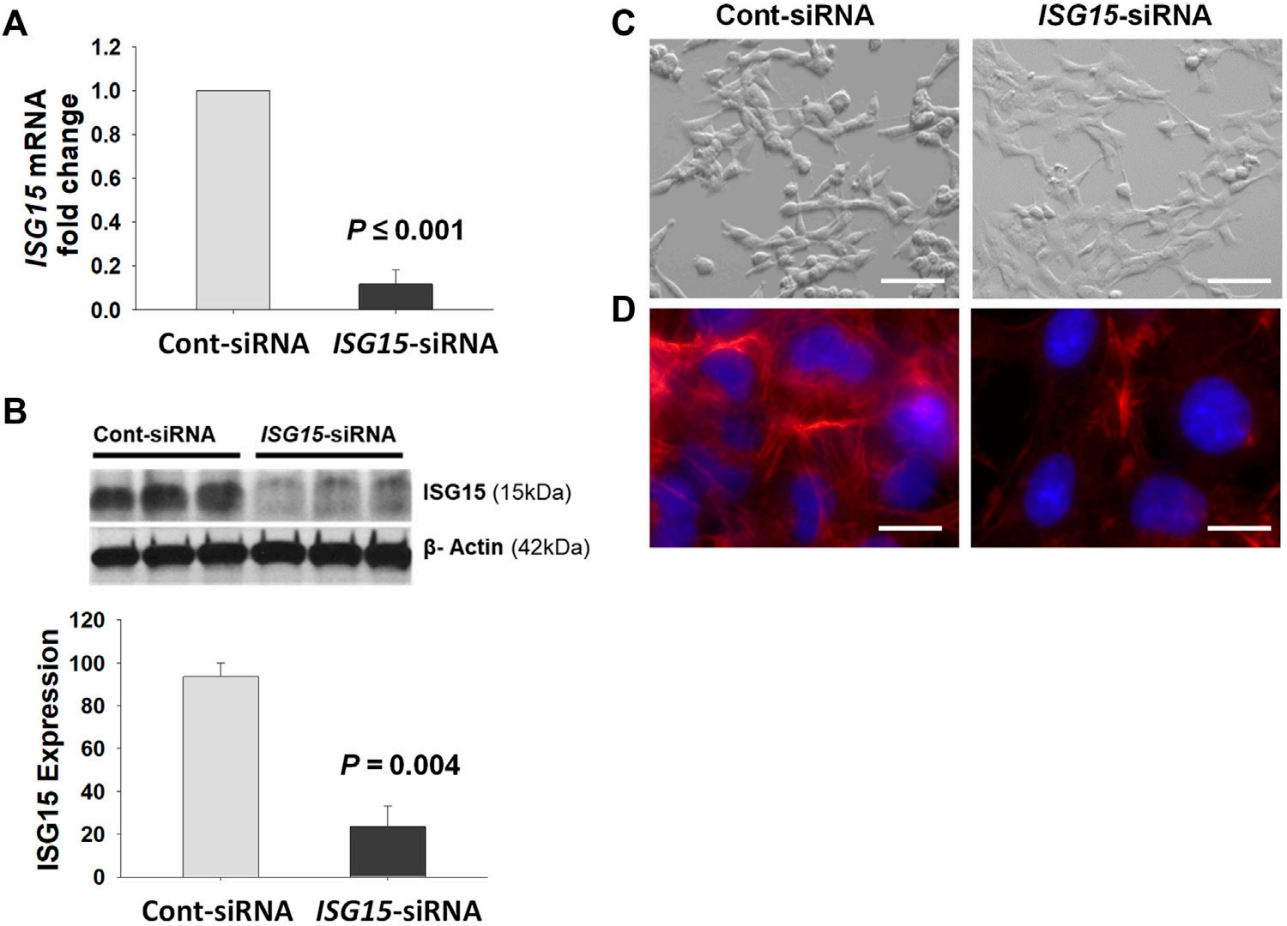
Decreased *ISG15* expression alters HTR8/SVneo cell morphology and reduces F- actin levels. **(A–D)** *ISG15* mRNA **(A)** and protein levels **(B)** as well as cell morphology **(C)** and TRITC-conjugated Phalloidin (red) fluorescence stained F-actin levels **(D)** are represented in *ISG15*-siRNA vs. control- siRNA transfected HTR8/SVneo cells. Bars represent Mean ± SEM and compared by using *t*-test, *n* = 3, Scale bars = 30 µm **(C)**, Scale bars = 6 µm **(D).**

HTR8/SVneo cells transfected with *ISG15* siRNA displayed significant reduction in *ISG15* mRNA and protein levels by q-PCR and Western blot analysis, respectively (Figures 5A,B). Furthermore, compared to scramble siRNA (control) treatment, cells in the *ISG15* silenced group exhibited substantial cell morphology alterations of flattened/thin cell shape and firm attachment (Figure 5C), prompting us to assess levels of F-actin which is associated with cell morphology and migration (Gardel et al., 2010). Analysis of phalloidin fluorescence staining revealed less F-actin levels in *ISG15* silenced group vs. control siRNA (Figure 5D).

### *ISG15* siRNA Silencing Reduces Migration, Invasion, and Proliferation of HTR8/SVneo Cells

**FIGURE 6:**
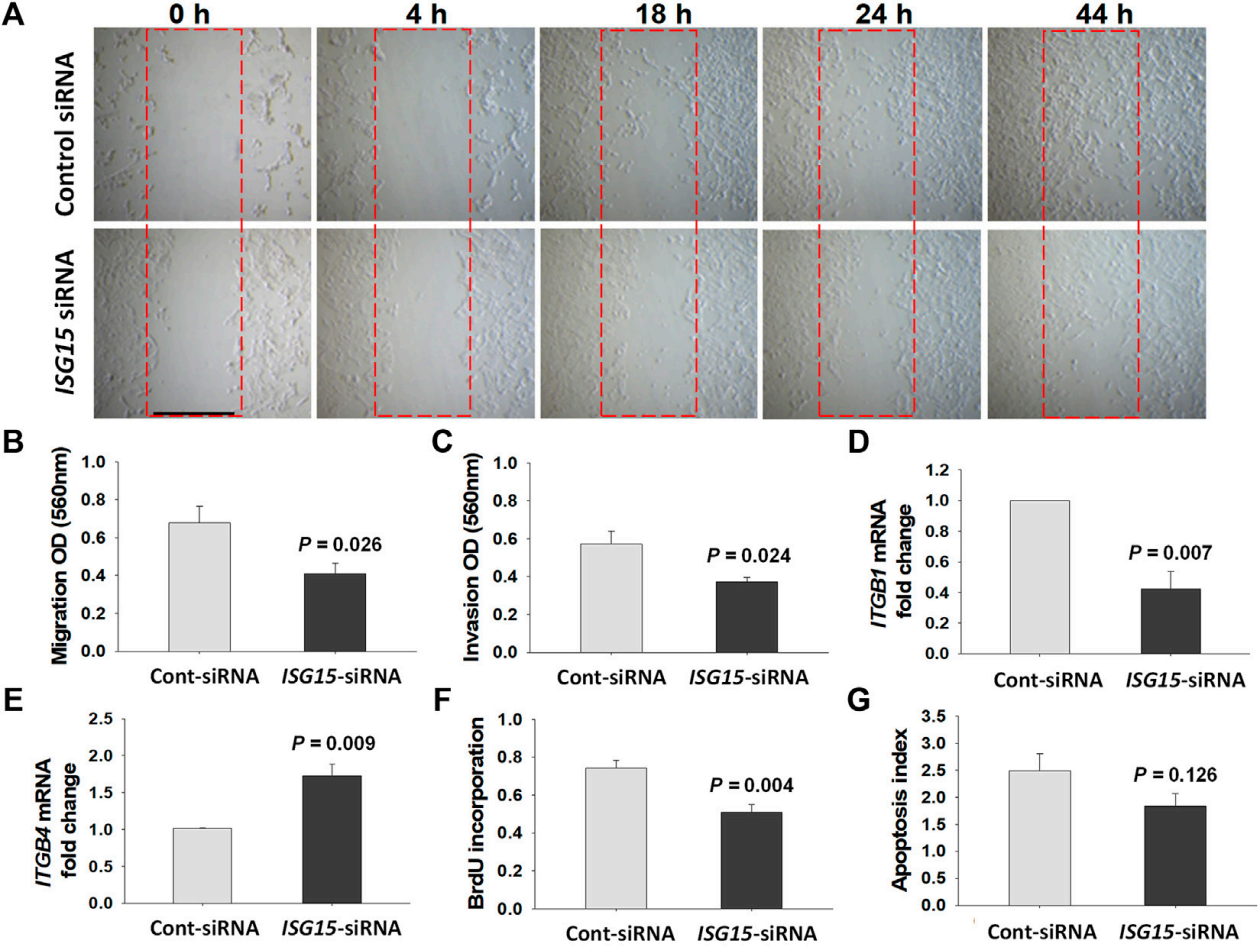
*ISG15* silencing reduces trophoblast migration, invasion and proliferation capacities. **(A)** *In vitro* wound healing (0–44 h), **(B)** migration (*n* = 5, 48 h), and **(C)** invasion (*n* = 5, 60 h) capacities, mRNA levels of **(D)** *ITGB1* (*n* = 3), **(E)** *ITGB4* genes (*n* = 3), as well as **(F)** proliferation (*n* = 5, 48 h) and **(G)** apoptotic index (*n* = 6, 48 h) are compared in control- vs. *ISG15*-siRNA transfected HTR8/SVneo cells. Bars represent Mean ± SEM and compared by using *t*-test. Scale bar = 500 µm.

Initial wound healing assay of HTR8/SVneo cells revealed a delayed wound closure following *ISG15*-siRNA versus control-siRNA treatment indicating reduced migration following *ISG15* silencing (Figure 6A). To confirm these results, we performed cell migration and invasion assays and found that compared to control-siRNA treatment, *ISG15*-siRNA treatment significantly reduced HTR8/SVneo cell migration (0.61 ± 0.06 vs. 0.36 ± 0.02, *p =* 0.003, Figure 6B **)** and invasion (0.57 ± 0.06 vs. 0.37 ± 0.02, *p =* 0.024, Figure 6C). For a mechanistic interpretation of the decreased migration and invasion in *ISG15* silenced trophoblasts, we then examined HTR8/SVneo cells for mRNA levels of integrin β1 (*ITGB1*, predominantly expressed by invasive trophoblasts) and integrin β4 (*ITGB4*, hemi-desmosomal adhesion marker predominantly expressed by villous trophoblasts) (Aplin, 1993; Damsky et al., 1994). These q-PCR analyses revealed that compared to control-siRNA treatment, *ISG15*-siRNA silencing significantly inhibited *ITGB1* expression (1.00 ± 0.00 vs. 0.42 ± 0.11, *p =* 0.007, Figure 6D) and enhanced *ITGB4* expression (1.01 ± 0.01 vs. 1.73 ± 0.15, *p =* 0.009, Figure 6E). Moreover, compared to control siRNA, *ISG15* silencing significantly reduced BrdU incorporation levels (0.74 ± 0.04 vs. 0.51 ± 0.04, *p =* 0.004, Figure 6F) without a significant alteration in apoptotic index (2.49 ± 0.31 vs. 1.84 ± 0.24, *p =* 0.126, Figure 6G).

### Reduced *ISG15* Levels Elevate Pro-inflammatory Cytokine Levels in EVTs

**FIGURE 7:**
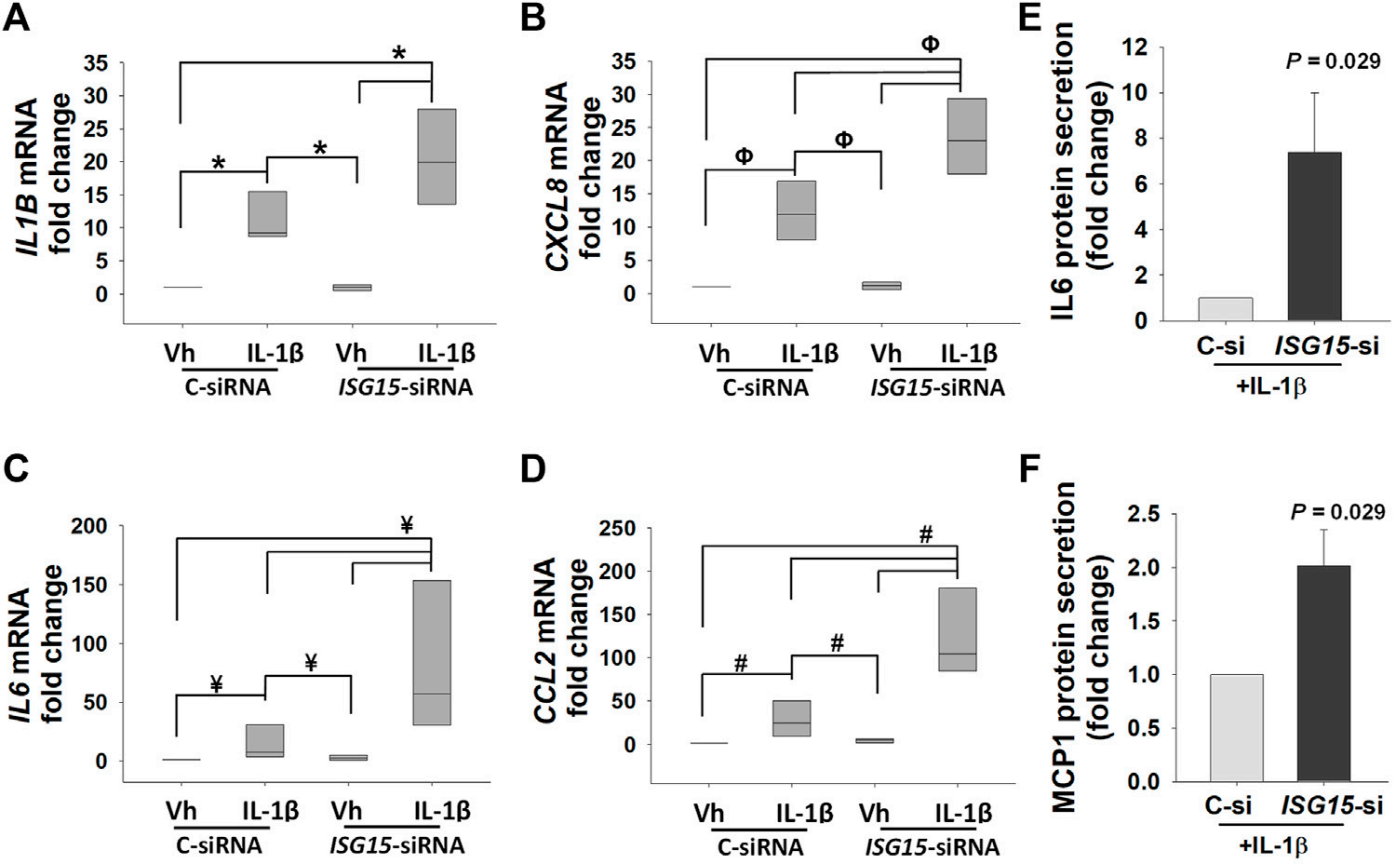
*ISG15* silencing amplifies IL-1β induced expression of pro-inflammatory cytokines. **(A–D)** Expression levels of *IL1B*, *CXCL8*, *IL6*, and *CCL2* mRNA in control vs. *ISG15*-siRNA transfected HTR8/SVneo cells, followed by 6 h treatment with vehicle (vh) or 10 ng/ml IL1β (IL-1β). **(E,F)** IL-6 and MCP-1, aka *CCL2*, protein secretion levels in *ISG15*-siRNA vs. control transfected HTR8/SVneo cells treated with 10 ng/ml IL1β (IL-1β). **(A–D)**: Bars represent median values and compared by using One way ANOVA, *n* = 4, *p = 0.007, ^Φ^ *P =* 0.005, ^¥^ *p =* 0.008, ^#^ *p =* 0.003. **(E,F)**: Bars represent Mean ± SEM and compared by using *t*-test (*n* = 4).

To uncover *ISG15* contribution in regulation of pro-inflammatory cytokines in EVTs, control- and *ISG15*-siRNA treated HTR8/SVneo cells were incubated with IL-1β, a pro-inflammatory cytokine implicated in the pathogenesis of PE (Lockwood et al., 2006; Lockwood et al., 2010). In control-siRNA transfected cells, IL-1β treatment significantly upregulated *IL1B* (9.2-fold, Figure 7A), *CXCL8* (12-fold, Figure 7B), *IL6* (7.5-fold Figure 7C), and *CCL2* (25.1-fold, Figure 7D) expressions. However, IL-1β treatment of HTR8/SVneo cells following silencing of *ISG15* resulted in an additional 1.9-, 7.7- and 4.2-fold higher *CXCL8*, *IL6*, and *CCL2* levels, respectively, compared to control–siRNA group treated with IL-1β (Figures 7B–D). ELISA analysis in HTR8/SVneo culture media supernatants confirmed that 10 ng/ml IL-1β treatment significantly increases IL-6 (Figure 7E **)** and MCP-1 (Figure 7F **)** protein secretion in *ISG15*-silenced group versus control group.

## Discussion

Normal pregnancy is accompanied by a mild inflammatory response within the uterine environment whereas PE is linked to a strong local and systemic inflammatory milieu due to altered immune cell types and numbers as well as elevated oxidative (Mannaerts et al., 2018; Tenorio et al., 2019) accompanying increased pro-inflammatory and decreased anti-inflammatory cytokine levels (Dimitriadis et al., 2005; Lockwood et al., 2013). Reduced differentiation, proliferation and/or limited decidual invasion of EVTs result in incomplete spiral artery transformation, which contributes to relatively reduced uteroplacental blood flow. The latter ultimately promotes the release of anti-angiogenic factors by the placenta that trigger maternal microvascular disease, hypertension, proteinuria, increased platelet turnover and the other features of the disease (Ozdemirci et al., 2016; Huppertz, 2018; Hu et al., 2021). EVTs express IL-6R, IL-11R and their common receptor gp130 (Nishino et al., 1990; Kojima et al., 1995; Paiva et al., 2007) indicating that at the maternal fetal interface EVTs are a principal paracrine target of increased decidual cell released IL-6 and IL-11 in PE (Lockwood et al., 2008; Basar et al., 2010). This may likely affect net transcriptional activities of these cytokines by one impacting the action of the other. Microarray results of this study support this premise that co-existence of IL-6 and IL-11 impairs the global transcription repertoire of trophoblasts compared to the presence of IL-11 alone, most probably through a mechanism that partial or complete IL-6 binding to gp130 blocks IL-11 action. This is also supported by the Venn diagram analysis of our microarray results (Supplementary Figure S4) that depicts differential gene expression among the treatment groups. Thus, differential expression of 76 genes (51 upregulated and 25 downregulated) were specific to IL-11 treatment; 25 genes (11 upregulated and 14 downregulated) were regulated by only IL-6 treatment; and 54 genes (22 upregulated and 32 downregulated) were regulated by only the combination of IL-11and IL-6 (Supplementary Figure S4).

Importantly, *ISG15* is the only commonly affected gene and is downregulated by IL-6 and IL-11 co-treatment compared to control or treatment with either IL-11 or IL-6 alone in microarray results, suggesting its involvement in PE. The *ISG15* gene, induced by type I IFNα/β, encodes a ubiquitin like 15 kDa protein which forms conjugates with a diverse pool of target proteins in a process known as ISGylation, a series of enzymatic reactions similar to that occur in ubiquitination (Dos Santos and Mansur, 2017). *ISG15* is involved in several anti-viral processes such as regulation of viral replication, proteasomal activity, autophagy, cytokine secretion, etc. (Sandy et al., 2020). In humans, homozygous loss of functional mutations in the *ISG15* gene, results in suppression of IFN-γ production in lymphocytes and cause severe auto-inflammation by inducing an abnormally hyper-IFN-α/β mediated immune response (Zhang et al., 2015).

Induction of endometrial *ISG15* expression in response to type I IFNs (IFN-α and IFN-τ) produced by implanting embryos has been reported in several species (Zhao et al., 2017), including human, baboon (Bebington et al., 1999), cow (Austin et al., 1996), sheep (Johnson et al., 1999), and mouse (Austin et al., 2003). As an early response to pregnancy, increased endometrial levels of *ISG15* in equine has been implicated as one of the repertoire of molecules associated with endometrial receptivity (Klein et al., 2011). Studies in *Isg15* deficient mice found that *Isg15* expression is required for pregnancy maintenance and/or fetal viability (Henkes et al., 2015). The lack of altered *ISG15* expression in decidual tissues of pseudo-pregnant mice (Bany and Cross, 2006) indicate that increased *ISG15* is mainly regulated by or released from developing embryos. However, the exact mechanism for *ISG15* involvement in implantation and maintenance of pregnancy is not clear yet. Our *in vivo* results demonstrating EVT specific expression of *ISG15* among other trophoblast types in the first and third trimester placental specimens indicate *ISG15* involvement in crucial EVT functions e.g., proliferation, invasion, etc. Increased *ISG15* expression in TS-EVTs, also link *ISG15* to the process of EVT differentiation. However, further studies are required to clarify cause/effect relationship between increased *ISG15* and EVT differentiation.

Reduced EVT specific *ISG15* expression may contribute to the pathogenesis of PE since our *in-situ* results revealed significantly lower *ISG15* immunoreactivity levels in EVTs, but not in decidual cells at the maternal-fetal interface of PE vs. GA-matched normal placental specimens. In a previous study, consistent with our findings, it has also been shown that both invasive trophoblasts and decidual cells express *ISG15* in human placenta. Moreover, this group also found a strong *ISG15* expression at CTB progenitor cells in the placental villi and cell column at the first trimester, but not in term placentas (Schanz et al., 2014). Our Western blot results confirm decidua basalis specific downregulation of *ISG15* in PE, which is verified by similar HLA-G expression between groups. In addition, since we didn’t observe a difference in maternal serum *ISG15* levels, downregulation of *ISG15* levels in the maternal-fetal interface in preeclampsia can be linked to a local dysregulation rather than a systemic impact.

Our *in-situ* findings of lower *ISG15* expression levels in preeclamptic extravillous trophoblasts together with pregnancy-specific endometrial *ISG15* expression (Joyce et al., 2005) suggest that *ISG15* expression is required for decidual invasion and uterine spiral artery remodeling. HTR8/SVneo cell line derived from first-trimester chorionic villi explant outgrowths and commonly used as a model for migration/invasion studies for invasive EVT characteristics (Canfield et al., 2019; Walker et al., 2021). Our *in vitro* findings demonstrating reduced migration and invasion in HTR8/SVneo cell cultures following *ISG15* silencing support our hypothesis that reduced *ISG15* expression is associated with decreased trophoblast invasion capacity, which impairs spiral artery transformation and triggers PE development.

*ISG15*-mediated mechanism(s) of EVT invasion is unknown. Integrins are the major cell surface receptors mediating cell-extracellular matrix (ECM) interaction (Venter et al., 2001) and contribute to EVT invasion (Damsky et al., 1994; Aplin et al., 1999). During implantation and placental development, CTBs gain an invasive phenotype that is accompanied by a switch in their integrin expression where α6β4 integrin expression diminishes and that α5β1 and α1β1 integrin expression enhances (Fisher et al., 1989; Damsky et al., 1992; Aplin, 1993; Cross et al., 1994). A previous study also reported failure of α1/β1 integrin upregulation and α6/β4 integrin downregulation in invasive CTBs in villous column and/or uterine wall in PE versus normal placental specimens (Zhou et al., 1993). Our *in vitro* analyses in *ISG15*-silenced HTR8/SVneo cells detected upregulation of β4 integrin and inhibition of β1 integrins and F-actin levels, providing a novel mechanism that *ISG15* modulates trophoblast invasion by alternating β1 and β4-integrin as well as F-actin levels.

In Isg15^−/−^ mice decidua, several mRNAs were differentially expressed many of which encode proteins that regulate survival and anti-apoptotic pathways, and the ultrastructural observations showed a disruption between decidual and trophoblastic cell interaction, which is further supported with degenerative structures detected in trophoblastic cells (Henkes et al., 2015). Same study concluded that *Isg15* deletion causes a primary lesion in decidua which may cause or be the consequence of lesions in the trophoblast cells during implantation. (Henkes et al., 2015). We also questioned putative roles of *ISG15* expression on trophoblast proliferation and apoptosis. Consistent with mentioned study, our BrdU incorporation and apoptosis assay results revealed that *ISG15* silencing in HTR8/SVneo decreases proliferation without affecting apoptotic index.

Increased decidual inflammation is strongly associated with the pathogenesis of PE (Bellos et al., 2018) and impaired trophoblast invasion (Rampersaud et al., 2020). This study also characterizes *ISG15* as critical regulator of pro-inflammatory cytokine expression in EVTs as confirmed by enhanced *IL6*, *IL1B*, *CXCL8*, and *CCL2* mRNA levels in *ISG15*-silenced HTR8/SVneo cells. Moreover, among these cytokines, *IL6* expression displayed the strongest response in *ISG15* silenced condition with a 7.7-fold increase, suggesting that a pathological cycle in which increased IL-6 inhibits *ISG15* levels that further increase IL-6 expression in EVTs. This observation is consistent with previous studies referring IL-6 as a key factor in PE, and suggested it as a biomarker of PE (Ferguson et al., 2017). These results indicate an anti-inflammatory effect of *ISG15* which is supported by previous studies that report *ISG15* inducing IL-10 expression in human primary monocytes (Dos Santos et al., 2018) and that humans with functional *ISG15* mutation developing exacerbated IFN-induced severe auto-inflammation (Zhang et al., 2015). As a limitation of our study, primary trophoblast cultures should be used in future studies to confirm our observation in HTR8/SVneo cell lines.

In summary, current findings indicate that 1) *in situ* expression of *ISG15* is restricted to EVT among several trophoblast types, 2) *in vitro* differentiation of TS cells toward EVTs is associated with increased *ISG15* expression, 3) *in situ* interstitial trophoblasts display significantly lower *ISG15* levels in PE, and 4) *in vitro* *ISG15* silencing results in altered morphology, reduced proliferation, and invasion as well as elevated pro-inflammatory cytokine expression in invasive trophoblastic cells. Collectively, these results demonstrate that *ISG15* is a critical regulator of EVT morphology and function and that reduced *ISG15* in EVTs is associated with PE likely by impairing trophoblast invasion into the decidua and enhancing local inflammation at the maternal-fetal interface. Thus, agents inducing *ISG15* expression may be a novel therapeutic approach in PE.

## Data Availability

The datasets presented in this study can be found in online repositories. The names of the repository/repositories and accession number(s) can be found below: https://www.ncbi.nlm.nih.gov/geo/, GSE171439.
