# Supplementary material for: Preeclampsia is Associated With Reduced ISG15 Levels Impairing Extravillous Trophoblast Invasion
Source: Front Cell Dev Biol. 2022 Jun 28;10:898088. doi: 10.3389/fcell.2022.898088 (PMC9274133; doi:10.3389/fcell.2022.898088)
Supplement: Supplementary file 1 [file Table1.DOCX]

**Supplementary Table.** List of genes which are ≥1.5-*fold* up- or down-regulated in CTB cultures by IL-11, IL-6, IL-11+ IL-6 treatment versus control**. FC:** Fold Change.

| **IL-11 *vs.* Control** | | | | **IL-6 *vs.* Control** | | | | **IL-11+IL-6 *vs.* Control** | | | |
| --- | --- | --- | --- | --- | --- | --- | --- | --- | --- | --- | --- |
| **Up-regulated Gene Symbols** | **FC** | **Down-regulated Gene Symbols** | **FC** | **Up-regulated Gene Symbols** | **F.C.** | **Down-regulated Gene Symbols** | **FC** | **Up-regulated Gene Symbols** | **FC** | **Down-regulated Gene Symbols** | **FC** |
| ***LMCD1*** | 3.92 | ***RPS4Y1*** | -12.30 | ***LMCD1*** | 3.43 | ***RPS4Y1*** | -10.92 | ***LMCD1*** | 4.11 | ***RPS4Y1*** | -8.28 |
| ***HS.572444*** | 3.13 | ***LOC100133662*** | -4.54 | ***CRH*** | 2.98 | ***LOC100133662*** | -3.57 | ***CRH*** | 3.33 | ***PRG2*** | -3.70 |
| ***IL8*** | 3.01 | ***EIF1AY*** | -3.83 | ***CYR61*** | 2.55 | ***PRG2*** | -3.38 | ***IL8*** | 2.71 | ***LOC100133662*** | -2.99 |
| ***CYR61*** | 2.94 | ***LAIR2*** | -2.76 | ***EGR1*** | 2.51 | ***EIF1AY*** | -2.92 | ***CYR61*** | 2.67 | ***LHB*** | -2.99 |
| ***CRH*** | 2.81 | ***LOC654191*** | -2.49 | ***VAMP5*** | 2.47 | ***LAIR2*** | -2.87 | ***HS.572444*** | 2.66 | ***CGB5*** | -2.92 |
| ***RASEF*** | 2.50 | ***GSTT1*** | -2.32 | ***IL8*** | 2.39 | ***CGB*** | -2.55 | ***TUBB3*** | 2.37 | ***CGB8*** | -2.86 |
| ***EGR1*** | 2.47 | ***PRG2*** | -2.29 | ***RASEF*** | 2.32 | ***LOC654191*** | -2.50 | ***RASEF*** | 2.33 | ***CGB1*** | -2.69 |
| ***VAMP5*** | 2.47 | ***LDHB*** | -2.22 | ***LOC643431*** | 2.28 | ***LEPREL1*** | -2.49 | ***HPGD*** | 2.31 | ***CGB*** | -2.67 |
| ***LOC441377*** | 2.33 | ***HBG2*** | -2.20 | ***HS.572444*** | 2.22 | ***LHB*** | -2.45 | ***EGR1*** | 2.28 | ***LAIR2*** | -2.65 |
| ***XIST*** | 2.29 | ***LEPREL1*** | -2.19 | ***B3GNT3*** | 2.22 | ***CGB8*** | -2.42 | ***B3GNT3*** | 2.26 | ***LOC654191*** | -2.45 |
| ***HLA-DMA*** | 2.26 | ***XDH*** | -2.17 | ***XIST*** | 2.18 | ***SERPINE2*** | -2.42 | ***C8ORF4*** | 2.20 | ***EIF1AY*** | -2.41 |
| ***NPFFR2*** | 2.17 | ***MAGEA4*** | -2.13 | ***NPFFR2*** | 2.13 | ***HIST1H4C*** | -2.39 | ***FOS*** | 2.19 | ***LEPREL1*** | -2.39 |
| ***B3GNT3*** | 2.14 | ***LCP1*** | -2.11 | ***TUBB3*** | 2.11 | ***CGB1*** | -2.38 | ***LOC643150*** | 2.17 | ***GPD1L*** | -2.36 |
| ***HS.554507*** | 2.13 | ***JARID1D*** | -2.09 | ***HLA-DMA*** | 2.11 | ***LDHB*** | -2.34 | ***HS.440518*** | 2.15 | ***GSTT1*** | -2.33 |
| ***TUBB3*** | 2.11 | ***TGFBI*** | -2.08 | ***PSG7*** | 2.10 | ***FSTL3*** | -2.30 | ***VAMP5*** | 2.15 | ***PRCP*** | -2.28 |
| ***RAB3B*** | 2.06 | ***GPC3*** | -2.06 | ***FOS*** | 2.08 | ***CGB5*** | -2.29 | ***LOC643431*** | 2.13 | ***LDHB*** | -2.24 |
| ***FAM118A*** | 2.05 | ***CGB1*** | -2.05 | ***RAB3B*** | 1.99 | ***GPD1L*** | -2.24 | ***ZNF83*** | 2.10 | ***HSPA1B*** | -2.19 |
| ***TRIP10*** | 2.01 | ***LOC151162*** | -2.05 | ***GTPBP4*** | 1.99 | ***PRCP*** | -2.22 | ***PSG7*** | 2.05 | ***GH1*** | -2.17 |
| ***CHURC1*** | 2.00 | ***LHB*** | -2.04 | ***LOC441377*** | 1.99 | ***GKN1*** | -2.20 | ***NPFFR2*** | 2.04 | ***FSTL3*** | -2.14 |
| ***CAMK2N1*** | 1.98 | ***FSTL3*** | -2.01 | ***HS.554507*** | 1.96 | ***GSTT1*** | -2.19 | ***HS.554507*** | 2.03 | ***XDH*** | -2.12 |
| ***ATG9B*** | 1.98 | ***HBG1*** | -1.98 | ***HS.440518*** | 1.93 | ***TGFBI*** | -2.12 | ***NFKBIZ*** | 2.03 | ***SERPINE2*** | -2.10 |
| ***LOC613037*** | 1.97 | ***SERPINE2*** | -1.98 | ***CAMK2N1*** | 1.92 | ***XDH*** | -2.11 | ***LOC643161*** | 2.03 | ***CSH1*** | -2.09 |
| ***LOC100190986*** | 1.96 | ***GKN1*** | -1.98 | ***TRIP10*** | 1.92 | ***LOC151162*** | -2.10 | ***HLA-DMA*** | 2.02 | ***LOC151162*** | -2.08 |
| ***PSG7*** | 1.96 | ***C7ORF33*** | -1.96 | ***HPGD*** | 1.92 | ***HBG2*** | -2.09 | ***CAMK2N1*** | 1.96 | ***LCP1*** | -2.07 |
| ***IL4R*** | 1.95 | ***GPD1L*** | -1.96 | ***FAM118A*** | 1.89 | ***LCP1*** | -2.05 | ***S100A9*** | 1.96 | ***TGFBI*** | -2.07 |
| ***ZNF83*** | 1.95 | ***PRCP*** | -1.96 | ***TAP1*** | 1.88 | ***GPC3*** | -2.01 | ***TRIP10*** | 1.95 | ***SLC19A3*** | -2.05 |
| ***TAP1*** | 1.92 | ***RRAGD*** | -1.96 | ***KRT8P9*** | 1.86 | ***HSPA1B*** | -2.01 | ***LOC441377*** | 1.95 | ***CSHL1*** | -2.04 |
| ***GTPBP4*** | 1.92 | ***HPCAL1*** | -1.94 | ***ZNF83*** | 1.83 | ***JARID1D*** | -2.00 | ***NUAK2*** | 1.94 | ***MAGEA4*** | -2.04 |
| ***KRT18P28*** | 1.90 | ***HIST1H4C*** | -1.94 | ***S100A9*** | 1.83 | ***HBG1*** | -1.93 | ***GTPBP4*** | 1.91 | ***ISG15*** | -1.98 |
| ***LOC149501*** | 1.90 | ***CGB8*** | -1.94 | ***NUAK2*** | 1.82 | ***ABP1*** | -1.92 | ***SH3BP4*** | 1.90 | ***GPC3*** | -1.97 |
| ***NUAK2*** | 1.89 | ***PSG1*** | -1.93 | ***C4ORF26*** | 1.81 | ***RPS4Y2*** | -1.91 | ***IL4R*** | 1.87 | ***GKN1*** | -1.97 |
| ***NAPRT1*** | 1.87 | ***CGB5*** | -1.92 | ***TRIM4*** | 1.80 | ***MAGEA4*** | -1.90 | ***VTCN1*** | 1.87 | ***HIST1H4C*** | -1.96 |
| ***STS-1*** | 1.87 | ***CGB*** | -1.91 | ***PDK4*** | 1.80 | ***PSG1*** | -1.89 | ***GFOD1*** | 1.86 | ***CSH2*** | -1.95 |
| ***ANKRD1*** | 1.87 | ***ZSWIM7*** | -1.91 | ***STS-1*** | 1.80 | ***UCK2*** | -1.88 | ***GPR177*** | 1.86 | ***MLLT1*** | -1.94 |
| ***FAM175A*** | 1.82 | ***MLLT1*** | -1.90 | ***KRT18P28*** | 1.80 | ***MLLT1*** | -1.87 | ***WDR18*** | 1.85 | ***MX1*** | -1.91 |
| ***LOC440993*** | 1.82 | ***RPL37A*** | -1.88 | ***IL4R*** | 1.79 | ***C7ORF33*** | -1.86 | ***XIST*** | 1.85 | ***HBG2*** | -1.90 |
| ***LOC647993*** | 1.81 | ***DNLZ*** | -1.87 | ***HS.553301*** | 1.79 | ***RRAGD*** | -1.85 | ***HS.553301*** | 1.84 | ***JARID1D*** | -1.89 |
| ***FOS*** | 1.80 | ***HSPA1B*** | -1.86 | ***STAT4*** | 1.78 | ***SLC19A3*** | -1.83 | ***KRT80*** | 1.84 | ***RPL14*** | -1.89 |
| ***SH3BP4*** | 1.80 | ***PAPPA*** | -1.85 | ***CHURC1*** | 1.78 | ***HPCAL1*** | -1.83 | ***KRT8P9*** | 1.84 | ***DFNA5*** | -1.86 |
| ***STAT4*** | 1.80 | ***SLC19A3*** | -1.84 | ***KRT80*** | 1.77 | ***RPL37A*** | -1.81 | ***ANKRD1*** | 1.83 | ***LOC100128936*** | -1.84 |
| ***SDC4*** | 1.80 | ***LOC100128936*** | -1.83 | ***LOC643150*** | 1.76 | ***ZSWIM7*** | -1.81 | ***STS-1*** | 1.83 | ***UCK2*** | -1.84 |
| ***LOC643431*** | 1.80 | ***LOC728492*** | -1.82 | ***SDC4*** | 1.76 | ***DNLZ*** | -1.80 | ***RAB3B*** | 1.81 | ***C7ORF33*** | -1.83 |
| ***LOC440353*** | 1.79 | ***UCK2*** | -1.81 | ***IFI44L*** | 1.76 | ***RPL14*** | -1.80 | ***CXCR6*** | 1.80 | ***RPL37A*** | -1.81 |
| ***LOC100128309*** | 1.79 | ***SLC9A3R1*** | -1.80 | ***CREG1*** | 1.74 | ***WNT7A*** | -1.78 | ***RARRES1*** | 1.79 | ***GPR172B*** | -1.81 |
| ***LOC729978*** | 1.79 | ***SLC40A1*** | -1.79 | ***SH3BP4*** | 1.73 | ***GLDC*** | -1.76 | ***FAM118A*** | 1.79 | ***DNLZ*** | -1.79 |
| ***HS.171171*** | 1.78 | ***CCDC109B*** | -1.73 | ***LOC653610*** | 1.73 | ***TMEM118*** | -1.76 | ***LOC149501*** | 1.79 | ***LOC402112*** | -1.78 |
| ***HS.553301*** | 1.78 | ***DFNA5*** | -1.73 | ***CCDC58*** | 1.72 | ***BSCL2*** | -1.75 | ***CD97*** | 1.78 | ***IFIT3*** | -1.77 |
| ***DUSP14*** | 1.78 | ***NUCB1*** | -1.70 | ***GPR177*** | 1.72 | ***SLC9A3R1*** | -1.74 | ***LOC647993*** | 1.78 | ***SLC9A3R1*** | -1.77 |
| ***SNAPC4*** | 1.77 | ***DOCK11*** | -1.69 | ***ANKRD1*** | 1.72 | ***LOC728492*** | -1.73 | ***SNAPC4*** | 1.75 | ***IFIT1*** | -1.76 |
| ***HS.535044*** | 1.77 | ***MFSD1*** | -1.69 | ***LOC149501*** | 1.71 | ***HTRA1*** | -1.71 | ***ZHX2*** | 1.74 | ***SLC40A1*** | -1.75 |
| ***LOC646753*** | 1.77 | ***NDRG2*** | -1.67 | ***NFKBIZ*** | 1.71 | ***SAMM50*** | -1.71 | ***LOC440993*** | 1.74 | ***BSCL2*** | -1.75 |
| ***CCDC58*** | 1.77 | ***RPRD2*** | -1.67 | ***RPL23AP53*** | 1.70 | ***SLC40A1*** | -1.69 | ***TRIM4*** | 1.73 | ***NDRG2*** | -1.75 |
| ***CASQ1*** | 1.77 | ***RPL14*** | -1.66 | ***C8ORF4*** | 1.70 | ***LOC100128936*** | -1.68 | ***ITGA2*** | 1.73 | ***WNT7A*** | -1.74 |
| ***STC2*** | 1.77 | ***ABP1*** | -1.66 | ***RGS2*** | 1.70 | ***GH1*** | -1.67 | ***HIST2H4A*** | 1.72 | ***SLC7A4*** | -1.73 |
| ***WDR18*** | 1.76 | ***FRZB*** | -1.65 | ***IQCG*** | 1.68 | ***TFCP2L1*** | -1.67 | ***RND3*** | 1.72 | ***IFIH1*** | -1.72 |
| ***PDK4*** | 1.76 | ***CYORF15A*** | -1.64 | ***WDR18*** | 1.68 | ***NDRG2*** | -1.67 | ***VNN3*** | 1.72 | ***TMEM118*** | -1.71 |
| ***TBC1D3G*** | 1.76 | ***ALDH9A1*** | -1.64 | ***COX7B2*** | 1.68 | ***AIM1*** | -1.67 | ***DUSP14*** | 1.71 | ***HLF*** | -1.71 |
| ***LOC23117*** | 1.74 | ***QDPR*** | -1.64 | ***FLJ10996*** | 1.68 | ***DFNA5*** | -1.67 | ***CDH5*** | 1.71 | ***HLA-G*** | -1.71 |
| ***C4ORF26*** | 1.73 | ***PKIB*** | -1.64 | ***GFOD1*** | 1.67 | ***SLC43A2*** | -1.66 | ***KRT18P28*** | 1.71 | ***SLC16A3*** | -1.70 |
| ***LOC643161*** | 1.72 | ***HMGB2*** | -1.64 | ***STC2*** | 1.67 | ***TREML2*** | -1.66 | ***LDHA*** | 1.70 | ***MFSD1*** | -1.69 |
| ***LOC644237*** | 1.72 | ***MARCKS*** | -1.63 | ***CRISPLD2*** | 1.67 | ***DOCK11*** | -1.66 | ***UGCG*** | 1.70 | ***HBG1*** | -1.69 |
| ***KRT80*** | 1.72 | ***HLF*** | -1.63 | ***LOC440993*** | 1.66 | ***SLC16A3*** | -1.66 | ***STAT4*** | 1.70 | ***PPIL3*** | -1.69 |
| ***ZHX2*** | 1.72 | ***SGCE*** | -1.63 | ***VTCN1*** | 1.65 | ***GPR172B*** | -1.65 | ***SDC4*** | 1.68 | ***IFI27*** | -1.68 |
| ***SDHAP2*** | 1.71 | ***TMEM118*** | -1.63 | ***VNN3*** | 1.65 | ***MFSD1*** | -1.65 | ***CASQ1*** | 1.67 | ***PIR*** | -1.68 |
| ***JUN*** | 1.71 | ***NUDT7*** | -1.62 | ***DUSP14*** | 1.65 | ***HLA-G*** | -1.64 | ***CDKN2AIP*** | 1.67 | ***CGB7*** | -1.67 |
| ***HS.440518*** | 1.70 | ***TMC4*** | -1.62 | ***CXCR6*** | 1.65 | ***CSHL1*** | -1.64 | ***HK2*** | 1.67 | ***SMPDL3A*** | -1.67 |
| ***FLNC*** | 1.69 | ***HS.568928*** | -1.62 | ***ZHX2*** | 1.64 | ***FRZB*** | -1.64 | ***CFLAR*** | 1.66 | ***HPCAL1*** | -1.67 |
| ***CMYA5*** | 1.69 | ***ENOSF1*** | -1.62 | ***HK2*** | 1.64 | ***DECR1*** | -1.63 | ***JUN*** | 1.65 | ***HERC5*** | -1.67 |
| ***RPL23AP53*** | 1.69 | ***SKAP2*** | -1.62 | ***LOC642934*** | 1.64 | ***SLC2A3*** | -1.63 | ***SLC16A2*** | 1.65 | ***GLDC*** | -1.66 |
| ***KRT8P9*** | 1.69 | ***DPYSL2*** | -1.62 | ***CDKN2AIP*** | 1.64 | ***MCM6*** | -1.63 | ***MIR205*** | 1.65 | ***HTRA1*** | -1.66 |
| ***TBC1D3I*** | 1.69 | ***RPS4Y2*** | -1.62 | ***L1TD1*** | 1.62 | ***HLF*** | -1.62 | ***SPINK1*** | 1.65 | ***ZSWIM7*** | -1.66 |
| ***LOC643150*** | 1.69 | ***SNAP23*** | -1.61 | ***LOC100129882*** | 1.62 | ***CCDC109B*** | -1.62 | ***GPNMB*** | 1.64 | ***AIM1*** | -1.65 |
| ***L1TD1*** | 1.69 | ***SLC2A3*** | -1.61 | ***RARRES1*** | 1.62 | ***CSH1*** | -1.61 | ***STC2*** | 1.64 | ***QDPR*** | -1.65 |
| ***LOC100132247*** | 1.69 | ***MYLIP*** | -1.61 | ***ITGA2*** | 1.61 | ***TJP3*** | -1.61 | ***LOC653610*** | 1.64 | ***GADD45G*** | -1.64 |
| ***C6ORF192*** | 1.68 | ***SUSD2*** | -1.61 | ***RAD21*** | 1.61 | ***LOC253039*** | -1.60 | ***MIR21*** | 1.64 | ***CCDC109B*** | -1.64 |
| ***TMEM106A*** | 1.68 | ***FASN*** | -1.60 | ***LOC647993*** | 1.61 | ***QDPR*** | -1.60 | ***40606.00*** | 1.64 | ***GSTA4*** | -1.64 |
| ***TMEM40*** | 1.68 | ***GLDC*** | -1.60 | ***SNAPC4*** | 1.61 | ***LOC402112*** | -1.59 | ***LOC729978*** | 1.64 | ***TREML2*** | -1.63 |
| ***AIM1L*** | 1.67 | ***WNT7A*** | -1.60 | ***KRT18P17*** | 1.60 | ***RPL8*** | -1.58 | ***C4ORF26*** | 1.64 | ***FRZB*** | -1.63 |
| ***GFOD1*** | 1.67 | ***LOC100131196*** | -1.58 | ***JUN*** | 1.60 | ***NUCB1*** | -1.58 | ***IQCG*** | 1.63 | ***RRAGD*** | -1.62 |
| ***LOC653610*** | 1.67 | ***RPL7*** | -1.58 | ***FLJ41603*** | 1.60 | ***TMEM16A*** | -1.58 | ***RGS2*** | 1.63 | ***TFCP2L1*** | -1.62 |
| ***CYLN2*** | 1.67 | ***TFCP2L1*** | -1.57 | ***CGA*** | 1.59 | ***ENOSF1*** | -1.58 | ***ATG9B*** | 1.63 | ***RPL8*** | -1.62 |
| ***DAPK3*** | 1.66 | ***TMEM140*** | -1.57 | ***LIMA1*** | 1.59 | ***CSH2*** | -1.57 | ***LOC642934*** | 1.63 | ***LOC642828*** | -1.61 |
| ***TRIML2*** | 1.66 | ***SLC43A2*** | -1.57 | ***HIST2H4A*** | 1.58 | ***FHDC1*** | -1.57 | ***LOC653506*** | 1.62 | ***FANCE*** | -1.61 |
| ***TRIM4*** | 1.66 | ***ANO2*** | -1.57 | ***LRP11*** | 1.58 | ***CHSY1*** | -1.57 | ***DDIT4*** | 1.62 | ***LOC642817*** | -1.61 |
| ***HK2*** | 1.66 | ***BSCL2*** | -1.57 | ***LOC645979*** | 1.58 | ***DPYSL2*** | -1.56 | ***TMEM40*** | 1.62 | ***DPYSL2*** | -1.60 |
| ***DUXAP3*** | 1.66 | ***LOC402112*** | -1.57 | ***LOC643161*** | 1.58 | ***SLC7A4*** | -1.56 | ***BCAR3*** | 1.62 | ***LAMB1*** | -1.60 |
| ***LOC100129650*** | 1.65 | ***GABRB1*** | -1.56 | ***RRP15*** | 1.57 | ***PIR*** | -1.56 | ***CREG1*** | 1.62 | ***ABP1*** | -1.60 |
| ***TMEM137*** | 1.65 | ***LOC253039*** | -1.56 | ***BTG2*** | 1.57 | ***ALDH9A1*** | -1.56 | ***STAT3*** | 1.61 | ***DECR1*** | -1.59 |
| ***SLC20A2*** | 1.65 | ***C13ORF27*** | -1.56 | ***HS.164221*** | 1.57 | ***CSGALNACT1*** | -1.56 | ***C7ORF40*** | 1.61 | ***HS.568928*** | -1.59 |
| ***FAM38A*** | 1.65 | ***HBA2*** | -1.56 | ***40606.00*** | 1.57 | ***GSTA4*** | -1.56 | ***L1TD1*** | 1.61 | ***RPS4Y2*** | -1.59 |
| ***GM2A*** | 1.65 | ***DNAJC15*** | -1.56 | ***ANXA3*** | 1.57 | ***GABRB1*** | -1.55 | ***CRISPLD2*** | 1.60 | ***C16ORF75*** | -1.58 |
| ***CREG1*** | 1.64 | ***CXORF57*** | -1.55 | ***LOC646753*** | 1.57 | ***LSM2*** | -1.55 | ***TAP1*** | 1.60 | ***BCL2*** | -1.58 |
| ***CRISPLD2*** | 1.64 | ***MCM6*** | -1.54 | ***LDHA*** | 1.56 | ***GHR*** | -1.55 | ***PDK4*** | 1.60 | ***SAMM50*** | -1.58 |
| ***ZNF738*** | 1.64 | ***MAN1A2*** | -1.54 | ***GM2A*** | 1.54 | ***TMC4*** | -1.54 | ***RPL23AP53*** | 1.60 | ***PSG1*** | -1.57 |
| ***WARS2*** | 1.63 | ***IFIH1*** | -1.54 | ***ATG9B*** | 1.54 | ***HS.568928*** | -1.54 | ***LOC147804*** | 1.60 | ***LOC388339*** | -1.57 |
| ***RGS2*** | 1.62 | ***SLC39A8*** | -1.54 | ***HS.170534*** | 1.54 | ***PAPPA*** | -1.54 | ***LOC440353*** | 1.59 | ***EPHB4*** | -1.57 |
| ***SLC25A34*** | 1.62 | ***SIAH1*** | -1.54 | ***CD97*** | 1.54 | ***MYLIP*** | -1.54 | ***OKL38*** | 1.59 | ***UBE2F*** | -1.57 |
| ***FLJ41603*** | 1.62 | ***BBS2*** | -1.53 | ***SPINK1*** | 1.54 | ***NUDT7*** | -1.53 | ***SLC20A2*** | 1.58 | ***TMED10*** | -1.56 |
| ***GPX3*** | 1.62 | ***NRP1*** | -1.53 | ***MIR21*** | 1.53 | ***MGC12965*** | -1.53 | ***LOC644237*** | 1.58 | ***GHR*** | -1.56 |
| ***LIMA1*** | 1.62 | ***UBE2F*** | -1.53 | ***TGFB2*** | 1.53 | ***CGB7*** | -1.53 | ***KRT18P17*** | 1.58 | ***LOC388556*** | -1.56 |
| ***FLJ10916*** | 1.61 | ***TMED10*** | -1.53 | ***LOC652846*** | 1.53 | ***AIF1L*** | -1.53 | ***C16ORF56*** | 1.57 | ***SUSD2*** | -1.56 |
| ***LOC653506*** | 1.61 | ***CSPP1*** | -1.53 | ***GADD45A*** | 1.52 | ***HMGB2*** | -1.53 | ***CNN3*** | 1.57 | ***TIMP1*** | -1.56 |
| ***RPS26L*** | 1.61 | ***SEC23B*** | -1.53 | ***LOC100129650*** | 1.52 | ***CHCHD10*** | -1.53 | ***COX7B2*** | 1.57 | ***LOC728492*** | -1.56 |
| ***MAP6D1*** | 1.60 | ***TREML2*** | -1.52 | ***GRAMD3*** | 1.52 | ***LOC652685*** | -1.53 | ***CEBPD*** | 1.57 | ***LOC651697*** | -1.56 |
| ***LOC389765*** | 1.60 | ***GH1*** | -1.52 | ***LOC650646*** | 1.52 | ***MBOAT7*** | -1.52 | ***MAP6D1*** | 1.57 | ***FASN*** | -1.55 |
| ***CXCR6*** | 1.60 | ***MTIF2*** | -1.52 | ***SLIT2*** | 1.52 | ***TTC39C*** | -1.52 | ***BTG2*** | 1.57 | ***BLVRA*** | -1.54 |
| ***LY6G6C*** | 1.59 | ***TBC1D9*** | -1.52 | ***XAGE1A*** | 1.52 | ***SUSD2*** | -1.52 | ***MGC16121*** | 1.57 | ***NPC2*** | -1.54 |
| ***MGC16121*** | 1.59 | ***SAMM50*** | -1.52 | ***P2RY6*** | 1.51 | ***ITFG2*** | -1.52 | ***ZCCHC8*** | 1.56 | ***NUDT7*** | -1.54 |
| ***FLJ10996*** | 1.59 | ***EPN3*** | -1.52 | ***SLC16A2*** | 1.51 | ***ZBED1*** | -1.51 | ***TGFB2*** | 1.56 | ***ANKRD6*** | -1.54 |
| ***C16ORF56*** | 1.58 | ***CHSY1*** | -1.51 | ***LOC644237*** | 1.51 | ***EML2*** | -1.51 | ***C17ORF96*** | 1.55 | ***LOC391833*** | -1.54 |
| ***KRT18P17*** | 1.58 | ***TMEM16A*** | -1.51 | ***STAT3*** | 1.51 | ***MAN1A2*** | -1.51 | ***RRP15*** | 1.55 | ***MCM6*** | -1.53 |
| ***DOCK5*** | 1.58 | ***ARMCX2*** | -1.51 | ***KRT17*** | 1.51 | ***BLVRA*** | -1.51 | ***C17ORF91*** | 1.55 | ***LOC100131196*** | -1.53 |
| ***ZNF486*** | 1.58 | ***PDGFRL*** | -1.51 | ***C7ORF40*** | 1.51 | ***KDELC2*** | -1.51 | ***XAGE1A*** | 1.55 | ***ATP6V0E2*** | -1.53 |
| ***SLC16A2*** | 1.58 | ***CSHL1*** | -1.51 | ***SNORD89*** | 1.51 | ***HMOX1*** | -1.51 | ***FLJ41603*** | 1.55 | ***ZBED1*** | -1.53 |
| ***GAPDH*** | 1.58 | ***PON2*** | -1.51 | ***SLC44A3*** | 1.50 | ***ARMCX2*** | -1.51 | ***KRT17*** | 1.55 | ***CDO1*** | -1.53 |
| ***40606.00*** | 1.57 | ***GSTA4*** | -1.51 | ***LOC729435*** | 1.50 | ***SLC25A5*** | -1.51 | ***HS.170534*** | 1.55 | ***ADHFE1*** | -1.53 |
| ***LOC728823*** | 1.57 | ***GPR172B*** | -1.51 | ***NAPRT1*** | 1.50 | ***JAM3*** | -1.51 | ***HS.164221*** | 1.54 | ***LY6E*** | -1.52 |
| ***ZNF682*** | 1.57 | ***BCL2*** | -1.51 | ***LOC340598*** | 1.50 | ***C7ORF44*** | -1.51 | ***FLNC*** | 1.54 | ***LOC646785*** | -1.51 |
| ***LOC648852*** | 1.57 | ***SLC16A3*** | -1.51 | ***UPP1*** | 1.50 | ***CPNE3*** | -1.51 | ***RAD21*** | 1.54 | ***SLC2A3*** | -1.51 |
| ***GADD45B*** | 1.57 | ***TTC39C*** | -1.51 | ***GPX3*** | 1.50 | ***WIPI1*** | -1.50 | ***TMEM206*** | 1.54 | ***LOC391370*** | -1.51 |
| ***IFI44L*** | 1.57 | ***MGC12965*** | -1.51 |  |  | ***SCNN1A*** | -1.50 | ***MYC*** | 1.54 | ***EPSTI1*** | -1.51 |
| ***C17ORF91*** | 1.57 | ***ANKRD57*** | -1.50 |  |  | ***RPS28*** | -1.50 | ***HS.535044*** | 1.53 | ***RPS28*** | -1.51 |
| ***LOC221981*** | 1.56 | ***SCNN1A*** | -1.50 |  |  | ***RPL7*** | -1.50 | ***TBC1D3G*** | 1.53 | ***CSGALNACT1*** | -1.51 |
| ***LOC440348*** | 1.56 | ***DNAJC27*** | -1.50 |  |  |  |  | ***LRP11*** | 1.53 | ***POMGNT1*** | -1.50 |
| ***ACOX3*** | 1.56 |  |  |  |  |  |  | ***LOC613037*** | 1.53 | ***C13ORF27*** | -1.50 |
| ***CFLAR*** | 1.55 |  |  |  |  |  |  | ***DUSP10*** | 1.53 | ***CHCHD10*** | -1.50 |
| ***LOC399965*** | 1.54 |  |  |  |  |  |  | ***SEMA3B*** | 1.53 | ***HMGB2*** | -1.50 |
| ***LOC644928*** | 1.54 |  |  |  |  |  |  | ***GRAMD3*** | 1.53 | ***RPL13A*** | -1.50 |
| ***SEMA3B*** | 1.54 |  |  |  |  |  |  | ***LOC100128309*** | 1.52 | ***RPL7A*** | -1.50 |
| ***LOC100132585*** | 1.54 |  |  |  |  |  |  | ***LOC100190986*** | 1.52 | ***LOC652685*** | -1.50 |
| ***IL10*** | 1.54 |  |  |  |  |  |  | ***ANXA3*** | 1.52 |  |  |
| ***ITGA2*** | 1.53 |  |  |  |  |  |  | ***MAP3K6*** | 1.52 |  |  |
| ***LOC727877*** | 1.53 |  |  |  |  |  |  | ***GADD45A*** | 1.52 |  |  |
| ***ZNF827*** | 1.53 |  |  |  |  |  |  | ***FLJ10996*** | 1.52 |  |  |
| ***LOC730990*** | 1.53 |  |  |  |  |  |  | ***LOXL4*** | 1.52 |  |  |
| ***GPR177*** | 1.53 |  |  |  |  |  |  | ***GPX3*** | 1.52 |  |  |
| ***COX7B2*** | 1.53 |  |  |  |  |  |  | ***FAM25A*** | 1.52 |  |  |
| ***ACIN1*** | 1.53 |  |  |  |  |  |  | ***GADD45B*** | 1.52 |  |  |
| ***C17ORF96*** | 1.53 |  |  |  |  |  |  | ***FLJ10916*** | 1.51 |  |  |
| ***MCM8*** | 1.52 |  |  |  |  |  |  | ***BCL6*** | 1.51 |  |  |
| ***LOC391075*** | 1.52 |  |  |  |  |  |  | ***SPNS2*** | 1.51 |  |  |
| ***IQCG*** | 1.52 |  |  |  |  |  |  | ***TBC1D3I*** | 1.51 |  |  |
| ***GRAMD3*** | 1.52 |  |  |  |  |  |  | ***LOC23117*** | 1.51 |  |  |
| ***WDR6*** | 1.52 |  |  |  |  |  |  | ***AGPAT5*** | 1.50 |  |  |
| ***HS.482960*** | 1.52 |  |  |  |  |  |  | ***TNFRSF1B*** | 1.50 |  |  |
| ***CENPT*** | 1.52 |  |  |  |  |  |  | ***HS.288735*** | 1.50 |  |  |
| ***ANGPTL4*** | 1.52 |  |  |  |  |  |  |  |  |  |  |
| ***LOC647987*** | 1.52 |  |  |  |  |  |  |  |  |  |  |
| ***LOC645979*** | 1.52 |  |  |  |  |  |  |  |  |  |  |
| ***DPRX*** | 1.52 |  |  |  |  |  |  |  |  |  |  |
| ***LOC647954*** | 1.52 |  |  |  |  |  |  |  |  |  |  |
| ***TLE6*** | 1.51 |  |  |  |  |  |  |  |  |  |  |
| ***GPNMB*** | 1.51 |  |  |  |  |  |  |  |  |  |  |
| ***HS.568058*** | 1.51 |  |  |  |  |  |  |  |  |  |  |
| ***NXF1*** | 1.51 |  |  |  |  |  |  |  |  |  |  |
| ***CPA4*** | 1.51 |  |  |  |  |  |  |  |  |  |  |
| ***HIST2H4A*** | 1.51 |  |  |  |  |  |  |  |  |  |  |
| ***XAGE1A*** | 1.50 |  |  |  |  |  |  |  |  |  |  |
| ***HS.513971*** | 1.50 |  |  |  |  |  |  |  |  |  |  |
