# Supplementary figures and images for: Preeclampsia is Associated With Reduced ISG15 Levels Impairing Extravillous Trophoblast Invasion
Source: Front Cell Dev Biol. 2022 Jun 28;10:898088. doi: 10.3389/fcell.2022.898088 (PMC9274133; doi:10.3389/fcell.2022.898088)

**Supplementary Figure 8** [
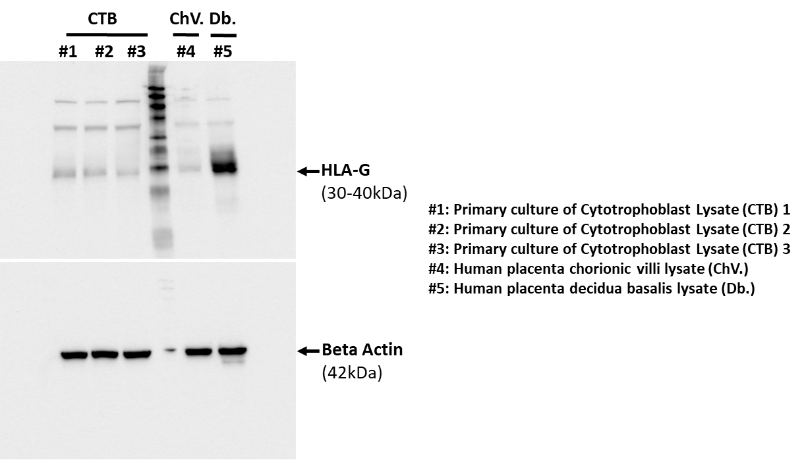
](HLA-G%20expression%20in%20%20primary%20culture%20of%20CTB.jpeg)

**Supplementary Figure 9.**


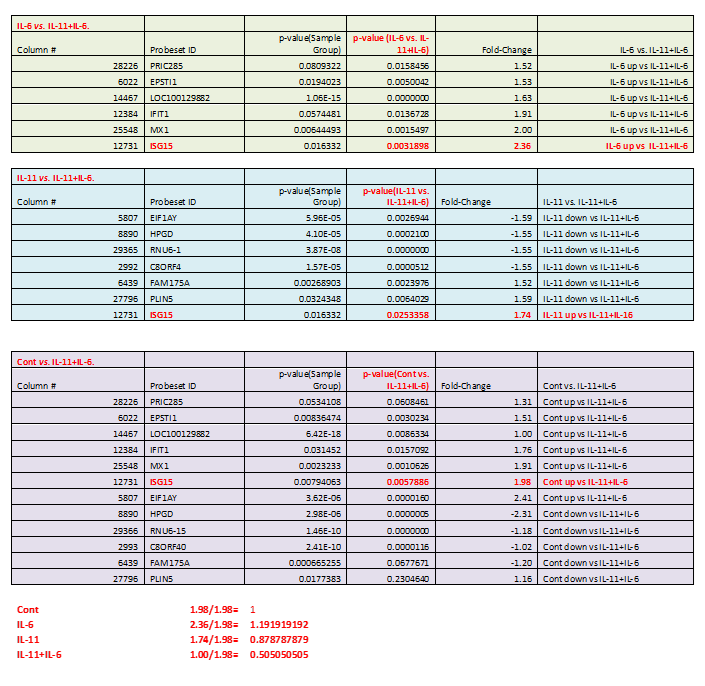

Supplement: Supplementary file 2 [file DataSheet2.docx]
