# Supplementary material for: Preeclampsia is Associated With Reduced ISG15 Levels Impairing Extravillous Trophoblast Invasion
Source: Front Cell Dev Biol. 2022 Jun 28;10:898088. doi: 10.3389/fcell.2022.898088 (PMC9274133; doi:10.3389/fcell.2022.898088)
Supplement: Supplementary file 3 [file DataSheet1.docx]

**Supplementary materials:**

**
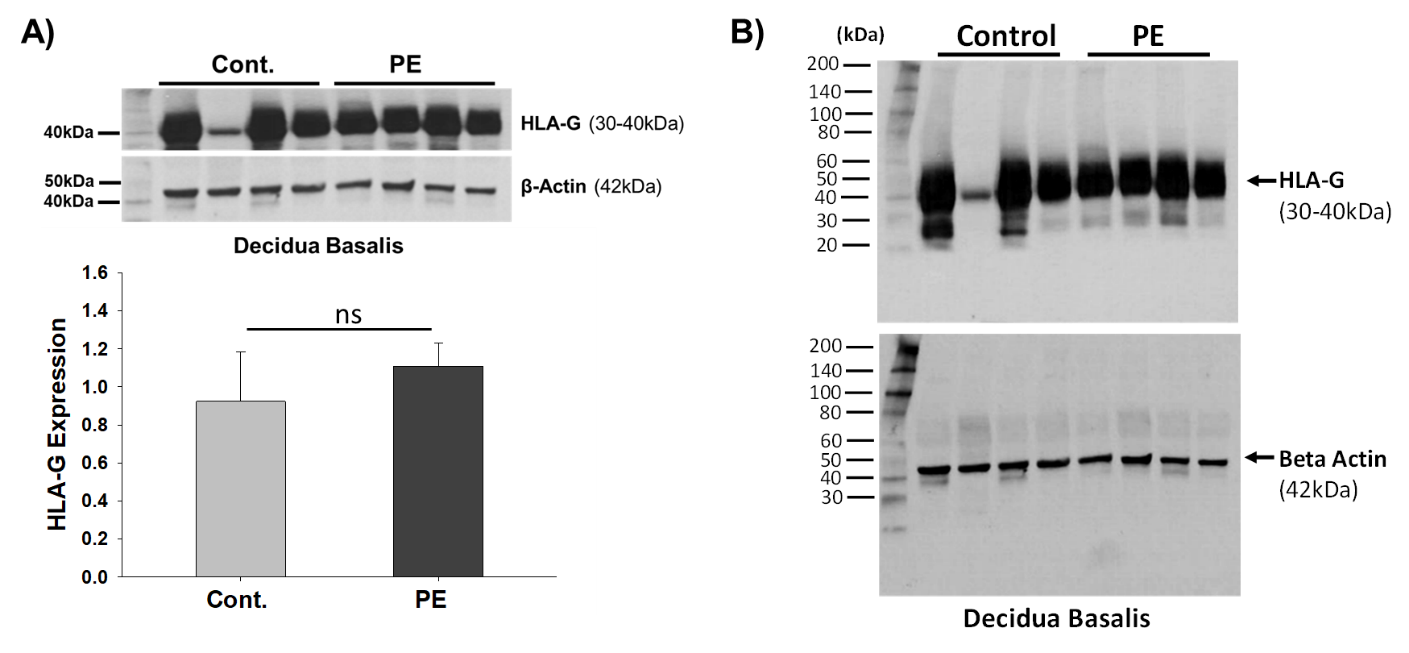
**

**Supplementary Figure 1**. **HLA-G expression confirms similar EVT cell numbers between groups.** Western blot analysis of decidua basalis shows similar HLA-G expression in Control (Cont.) *vs.* Preeclampsia (PE) specimens (**A**). Whole scan of Western Blots (**B**). Bars represent Mean± SEM and compared by using t-test, n=4/group, ns: not significant


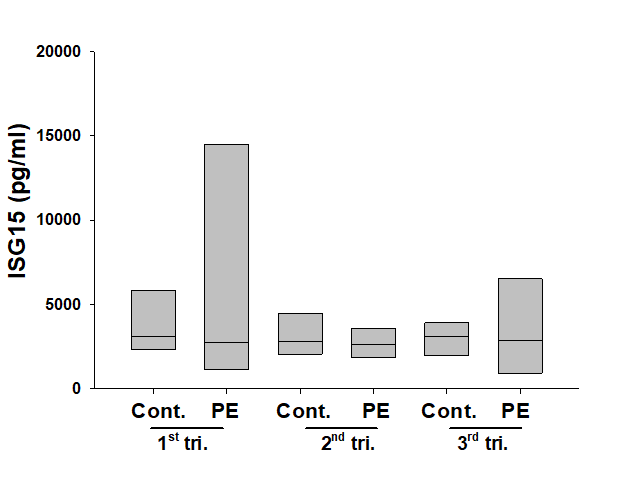


**Supplementary Figure 2. Serum ISG15 levels during pregnancy.** ISG15 serum levels did not display a significant difference among groups at any gestational age. Bars represent median values and compared by using Mann-Whitney U test, n=7/group. Cont: Control, PE: Preeclampsia, tri: Trimester


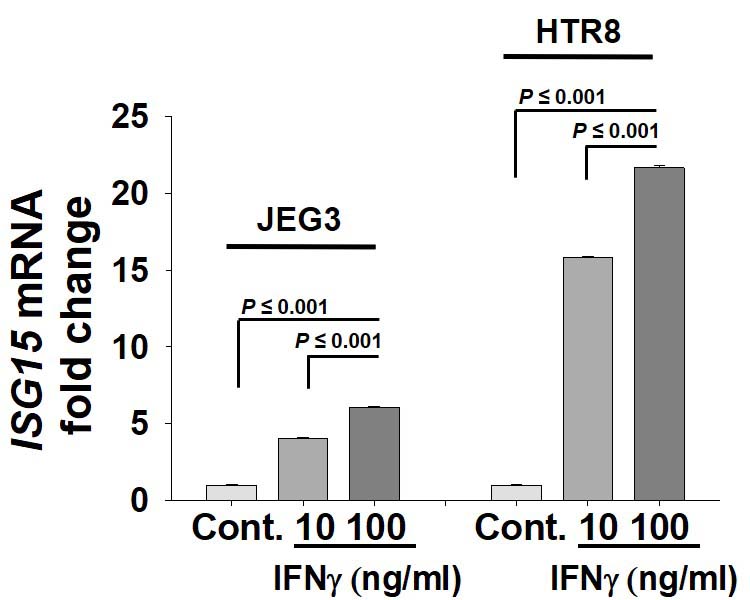


**Supplementary Figure 3. *ISG15* mRNA expression among cell lines.** *ISG15* mRNA expression in JEG3 and HTR8/SV^neo^ cell lines in response to 10-100ng/ml IFNγ treatment. Bars represent Mean± SEM and compared by using One way ANOVA, n=4.


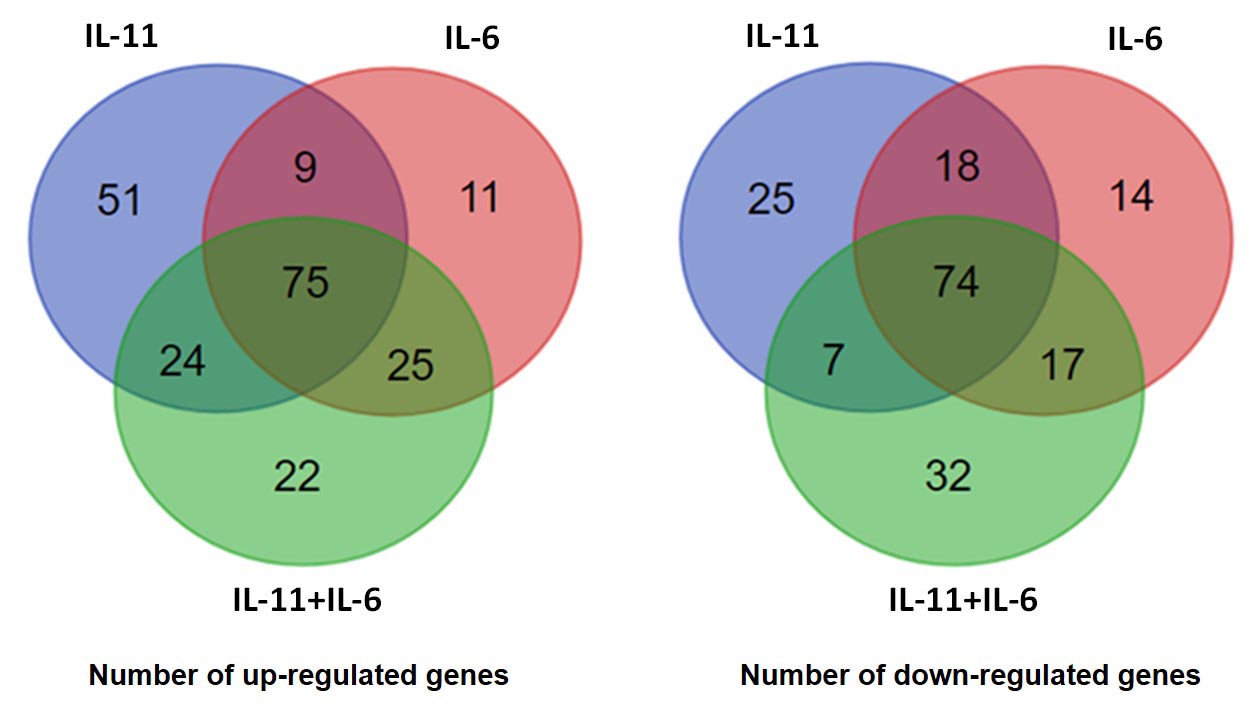


**Supplementary Figure 4.** Venn diagram illustrating distribution of number of genes differentially regulated commonly or specifically by treatment with IL-11 or IL-6 or IL-11+IL-6 *vs*. control in CTB cell cultures.


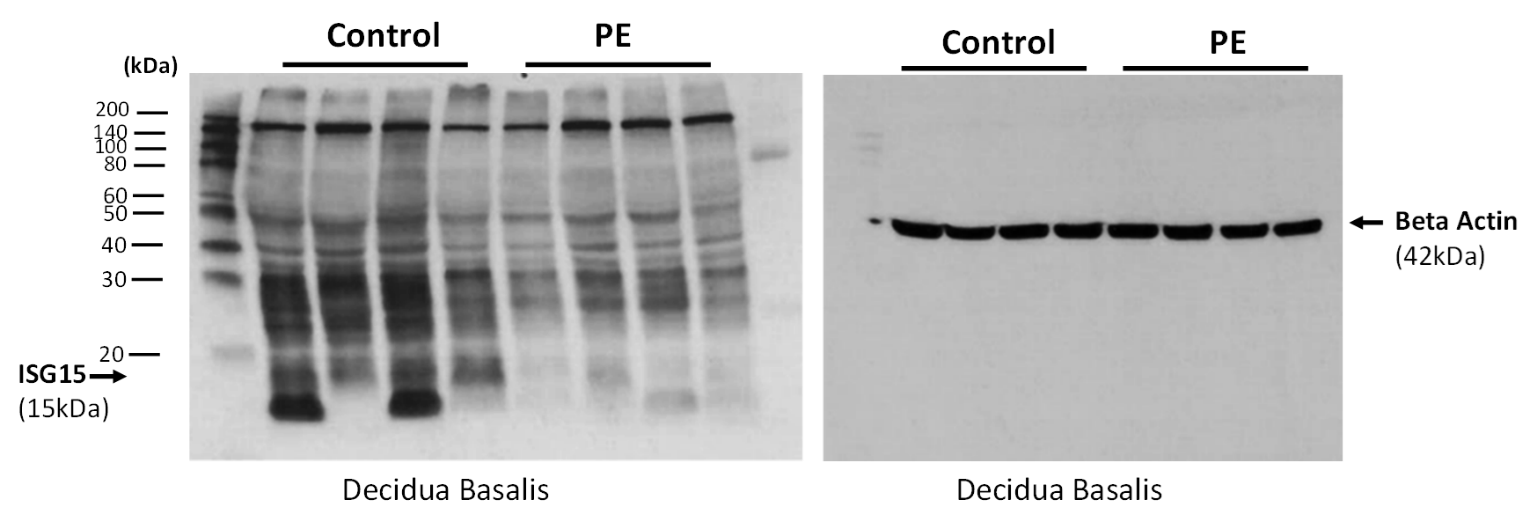


**Supplementary Figure 5.** Whole scan Western blotting results of ISG15 and corresponding Beta actin densities that represented in Figure **4C.**


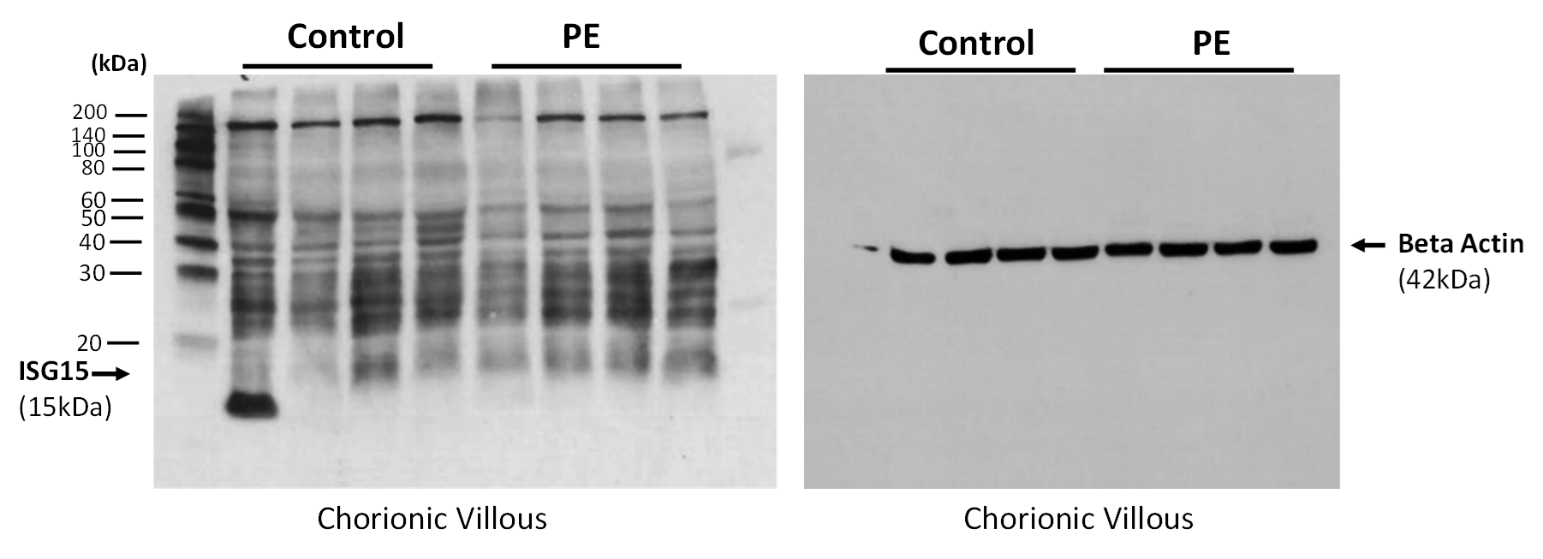


**Supplementary Figure 6.** Whole scan Western blotting results of ISG15 and corresponding Beta actin densities that represented in Figure 4D.

**
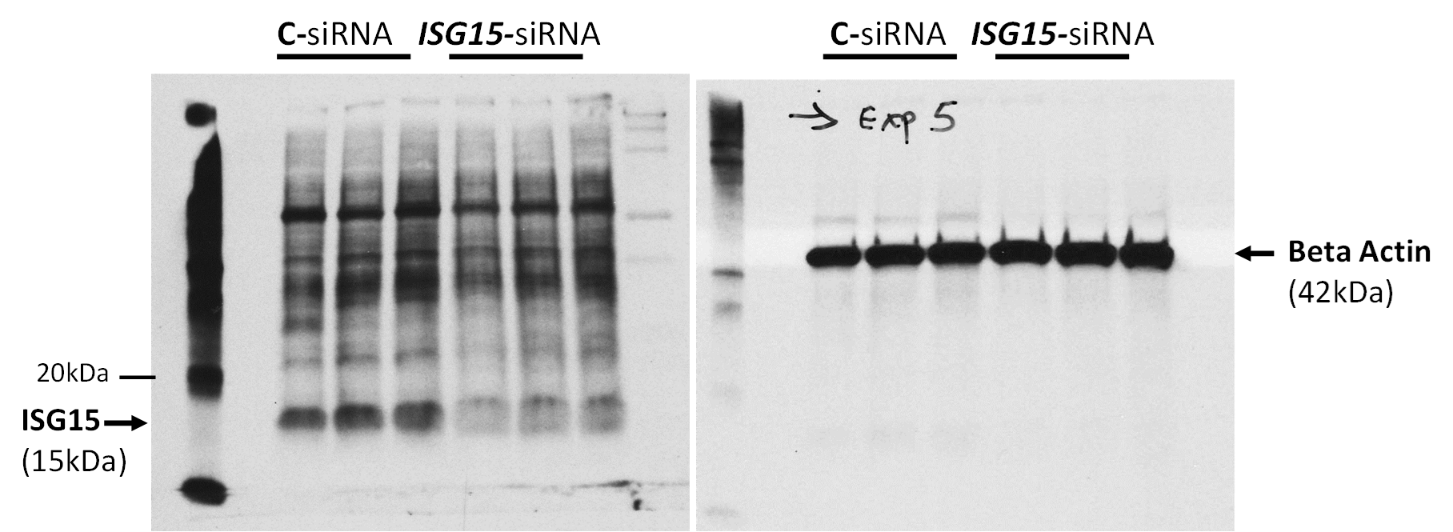
**

**Supplementary Figure 7.** Whole scan Western blotting results of ISG15 and corresponding Beta actin densities that represented in Figure 5B.
